# Soil microbiome manipulation triggers direct and possible indirect suppression against *Ralstonia solanacearum* and *Fusarium oxysporum*

**DOI:** 10.1038/s41522-021-00204-9

**Published:** 2021-04-12

**Authors:** Xuhui Deng, Na Zhang, Zongzhuan Shen, Chengzhi Zhu, Hongjun Liu, Zhihui Xu, Rong Li, Qirong Shen, Joana Falcao Salles

**Affiliations:** 1 Jiangsu Provincial Key Lab of Solid Organic Waste Utilization, Jiangsu Collaborative Innovation Center of Solid Organic Wastes, Educational Ministry Engineering Center of Resource-saving fertilizers, Nanjing Agricultural University, Nanjing, Jiangsu PR China; 2 The Key Laboratory of Plant Immunity, Nanjing Agricultural University, Nanjing, Jiangsu PR China; 3 Microbial Ecology Cluster, Genomics Research in Ecology and Evolution in Nature, Groningen Institute for Evolutionary Life Sciences, University of Groningen, Groningen, the Netherlands

**Keywords:** Microbiome, Pathogens

## Abstract

Soil microbiome manipulation can potentially reduce the use of pesticides by improving the ability of soils to resist or recover from pathogen infestation, thus generating natural suppressiveness. We simulated disturbance through soil fumigation and investigated how the subsequent application of bio-organic and organic amendments reshapes the taxonomic and functional potential of the soil microbiome to suppress the pathogens *Ralstonia solanacearum* and *Fusarium oxysporum* in tomato monocultures. The use of organic amendment alone generated smaller shifts in bacterial and fungal community composition and no suppressiveness. Fumigation directly decreased *F. oxysporum* and induced drastic changes in the soil microbiome. This was further converted from a disease conducive to a suppressive soil microbiome due to the application of organic amendment, which affected the way the bacterial and fungal communities were reassembled. These direct and possibly indirect effects resulted in a highly efficient disease control rate, providing a promising strategy for the control of the diseases caused by multiple pathogens.

## Introduction

Ecosystem management is important to ensure the long-term persistence of services. In agricultural systems, intensification leads to a decrease in microbial community diversity^1^, activity^2^ and suppressiveness^3^, potentially leading to an unbalanced proliferation of soil harmful microbes, including variety of notorious pathogens. In this context, practices that improve soil resistance or resilience to disease could potentially ensure high agricultural productivity, thus diminishing the environmental footprint of agriculture due to a reduction in pesticide use. However, turning a disease conducive into a suppressive soil requires robust strategies that are able to bypass the natural resistance of soil microbiome^4,5^. Thus, to manipulate the soil microbiome, it is important to generate specific environmental perturbations that affect the subsequent structuring of soil microbiome^4,5^.

In addition to the environmental disturbances experienced by many soil communities, disturbances often arise from different land use management regimes and associated practices^4^. Fumigation, a widely used practice for suppressing soil-borne diseases^6,7^, especially fungal pathogens^8^, represents a drastic disturbance to the soil microbiome^9,10^. However, fumigation often leads to transient disease suppression as soils experience rapid pathogen reinfestation^11^. Soil organic amendment is another widely accepted management strategy to create sustainable production and maintain ecosystem health^12^. The further complementation of organic amendments with biocontrol agents (bio-organic fertilizer) represents a promising strategy for the suppression of soil-borne diseases^13,14^. This approach is widely accepted in China, where bio-organic fertilizer application improves plant growth while protecting plant roots from soil-borne pathogens^15,16^. However, the efficiency of bio-organic fertilizer in suppressing soil-borne diseases is low in highly infested soils^10,17^ or soils infested by several pathogens, evoking the need for combined strategies when pathogen abundances in soils are excessive. For example, tomato usually suffers from *Fusarium* wilt disease caused by *Fusarium oxysporum*^18^, as well as from bacterial wilt disease caused by *Ralstonia solanacearum*^19^.

Although multiple pathogens associated with soil-borne diseases normally co-occur in natural environments^20,21^, most of the studies on disease suppression focus on a single pathogen, making the translation from experimental to natural conditions less likely to be successful, especially when pathogens respond differently to the applied practices. Common approaches such as the direct reduction of soil pathogenic microbial agents^22^ and indirect disease suppression induced by soil microbiome manipulation^23^ have been used to reduce several pathogens; however, the effectiveness of the approaches might depend on pathogen taxonomy, as the suppression mechanisms of fungal and bacterial pathogens differ. Furthermore, coinfection by bacterial and fungal pathogens can alter the outcome of the disease and favor the development of pathogens^24,25^. For instance, the interaction between *Rhizopus microsporus* and *Burkholderia* sp. can potentially lead to blight in rice seedlings through the secretion of a phytotoxin known as rhizoxin^26^. The distinct suppression mechanisms and potential interactions highlight the need to take a multi-pathogen approach when addressing soil-borne disease suppression.

We have previously found that continuous tomato planting leads to an increase in the incidence of two pathogens, *F. oxysporum* and *R. solanacearum*, whose inherent physiological and ecological differences hinder the use of a single disease control strategy with equal efficiency against both pathogens^27^. Here we propose to use a two-step approach, where the addition of bio-organic fertilizer is preceded by an initial disturbance of the soil microbiome through fumigation. Our hypothesis is that fumigation will decrease the soil microbial abundance, making it less resistant to invasion by the microbiome associated with bio-organic fertilizer. Moreover, we expect that the diversity of nutrients and the microbiome associated with bio-organic fertilizer will stimulate the growth of beneficial soil microbiomes, collectively leading to a suppressive microbiome capable of restricting disease incidence in severely infested soil. We tested this hypothesis in a tomato monoculture field where soils were amended with organic fertilizer (chicken manure compost) or bio-organic fertilizer (compost amended with *Bacillus* strain) and either fumigated or not, generating four treatments: un-fumigation + organic fertilizer; un-fumigation + bio-organic fertilizer; fumigation + organic fertilizer; fumigation + bio-organic fertilizer. We then followed the changes in pathogen abundance and shifts in bacterial and fungal communities before and after treatment, in both bulk and rhizosphere soils, given that plants greatly rely on their rhizosphere microbiome for uptake of nutrients and protection against pathogens^28^. Overall, these results will (i) deepen our understanding of how rhizosphere and bulk soil microbial assemblies respond to a two-step perturbation strategy, (ii) disentangle the general disease suppression mechanisms of the perturbation strategy in response to *F. oxysporum* and *R. solanacearum*, respectively, and (iii) verify to what extent disturbance can foster microbiome manipulations to achieve better ecosystem functions.

## Results

### Disease incidences among different treatments

**Fig. 1:**
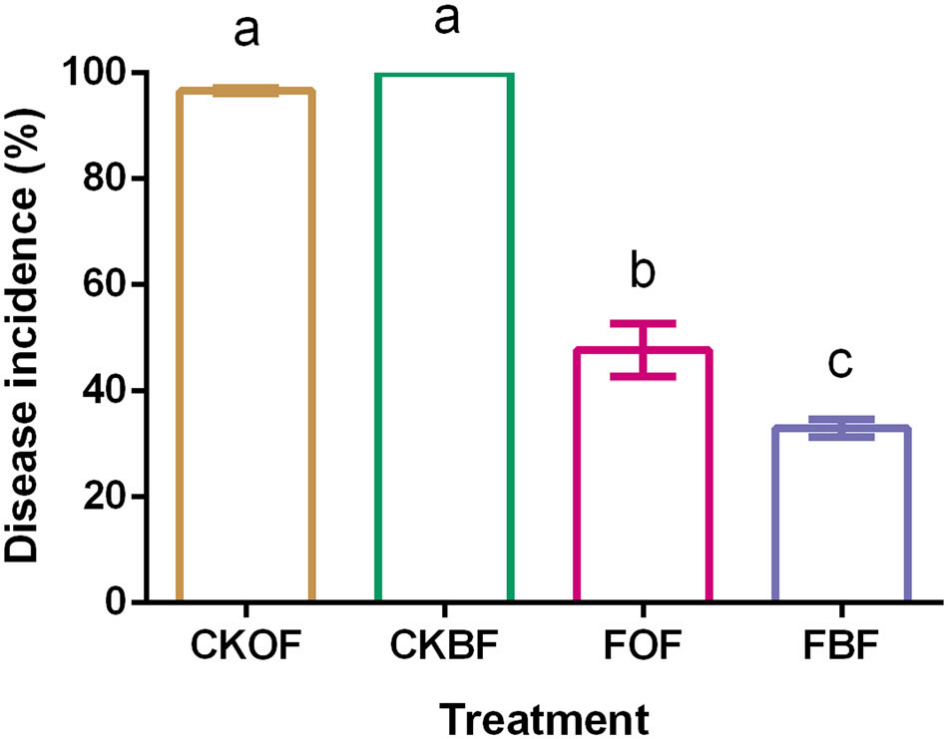
Results regarding wilt disease in tomato plants 3 months after transplantation in 2015 spring season. CKOF: organic fertilizer was amended in un-fumigated soil; CKBF: bio-organic fertilizer was amended in un-fumigated soil; FOF: organic fertilizer was amended in fumigated soil; FBF: bio-organic fertilizer was amended in fumigated soil. All values are the mean of three replicates and error bar represents the standard deviation of mean. Bars with different letters indicate significant differences among the four treatments as defined by Tukey’s test (*p* < 0.05).

Tomato wilt disease incidences among different treatments in each season were significantly lower (*F*_ANOVA_ = 188.8, *p* < 0.001; in Fig. 1) under fumigation treatments (FOF, FBF) with a value of 33–48% compared to the un-fumigated treatments (CKOF, CKBF; 97–100%). The results obtained in 2015 were consistent with those observed in spring 2014 (Supplementary Fig. 2). Moreover, variation partitioning analysis (VPA) revealed that fumigation contributed 100% of disease control, and different types of organic amendments only showed a 1.18% contribution (Supplementary Fig. 3). Due to difficulty in distinguishing the disease symptoms caused by each of the pathogens, and given that disease incidence is often significantly correlated with pathogen density^29,30^, in this study, we used the abundance of *R. solanacearum* and *F. oxysporum* based on qPCR to characterize the potential control effect of fumigation and bio-organic fertilizer.

### Pathogen abundances and their relationships among different compartments

**Fig. 2:**
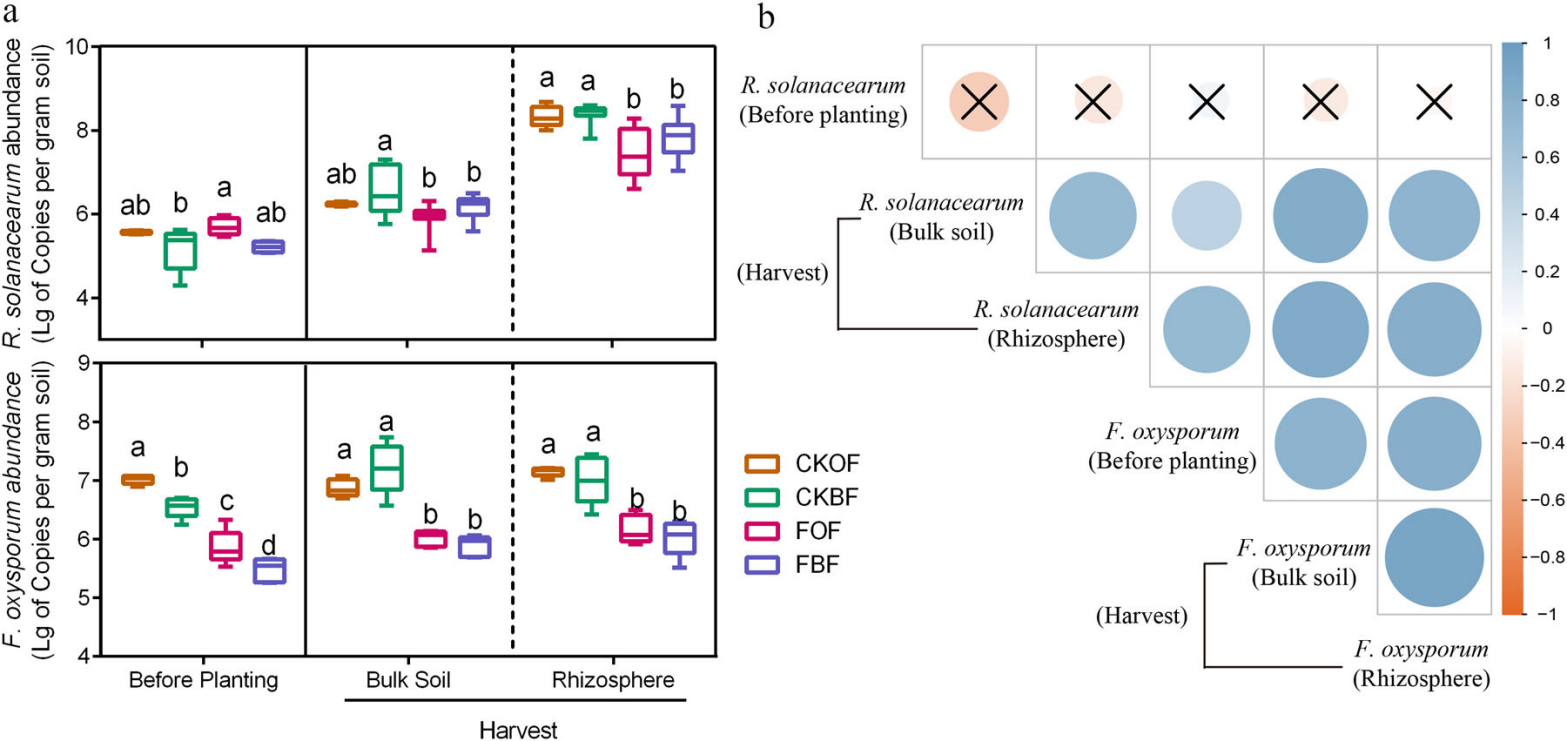
Quantitative PCR results for *R. solanacearum* and *F. oxysporum* in the bulk soil and rhizosphere. **a** Abundance of *R. solanacearum* and *F. oxysporum*. **b** Correlation between two kinds of pathogens’ abundant of different compartment. CKOF: organic fertilizer was amended in un-fumigated soil; CKBF: bio-organic fertilizer was amended in un-fumigated soil; FOF: organic fertilizer was amended in fumigated soil; FBF: bio-organic fertilizer was amended in fumigated soil. Different letters indicate significant differences among the four treatments as defined by Tukey’s test (*p* < 0.05).

Quantification of pathogen abundance through qPCR revealed a complex pattern depending on the pathogen. Regarding the abundance of *R. solanacearum*, we observed no significant differences between fumigated and un-fumigated soil samples before planting (*t*-test, *p* = 0.611), whereas significantly lower abundances were observed during harvest (both fumigation treatments FOF and FBF) in bulk (*t*-test, *p* = 0.005) and rhizosphere (*t*-test, *p* < 0.001) soil samples (Fig. 2a). For *F. oxysporum*, significantly lower abundances were observed in the fumigation treatments (FOF and FBF) before planting (*t*-test, *p* < 0.001), and this trend was sustained in bulk soil (*t*-test, *p* < 0.001) and the rhizosphere (*t*-test, *p* < 0.001) at harvest (Fig. 2a). Furthermore, no significant correlation of the abundance of *R. solanacearum* was observed between before transplanting and during harvest, while a significant relationship was observed for *F. oxysporum* (Fig. 2b). Finally, in the rhizosphere at harvest, the results of qPCR and MiSeq sequencing both showed that *R. solanacearum* (*p*_(abundance)_ < 0.001, *p*_(relative abundance)_ = 0.001) and *F. oxysporum* (*p*_(abundance)_ < 0.001, *p*_(relative abundance)_ < 0.001) were correlated with disease incidence (Supplementary Fig. 4). None of the soil properties measured affected the abundance of *R. solanacearum* and *F. oxysporum* (*p* > 0.05; Table 1).

**Table 1:** Partial correlation test between soil properties and abundance of two kinds of pathogens.

		Available potassium	Total potassium	Ammonium nitrogen	Nitrate	pH	Electrical conductance	Total phosphorus
*R. solanacearum*	*r*	−0.146	−0.252	0.188	−0.247	0.233	−0.198	−0.209
	*p*	0.38	0.127	0.258	0.135	0.159	0.232	0.208
*F. oxysporum*	*r*	−0.069	−0.164	−0.086	−0.03	0.222	−0.066	−0.052
	*p*	0.68	0.326	0.606	0.86	0.181	0.692	0.757

The effect of time and fumigation were removed by the partial correlation test.

### Microbial abundance, diversity, and their relationship with disease incidence

**Fig. 3:**
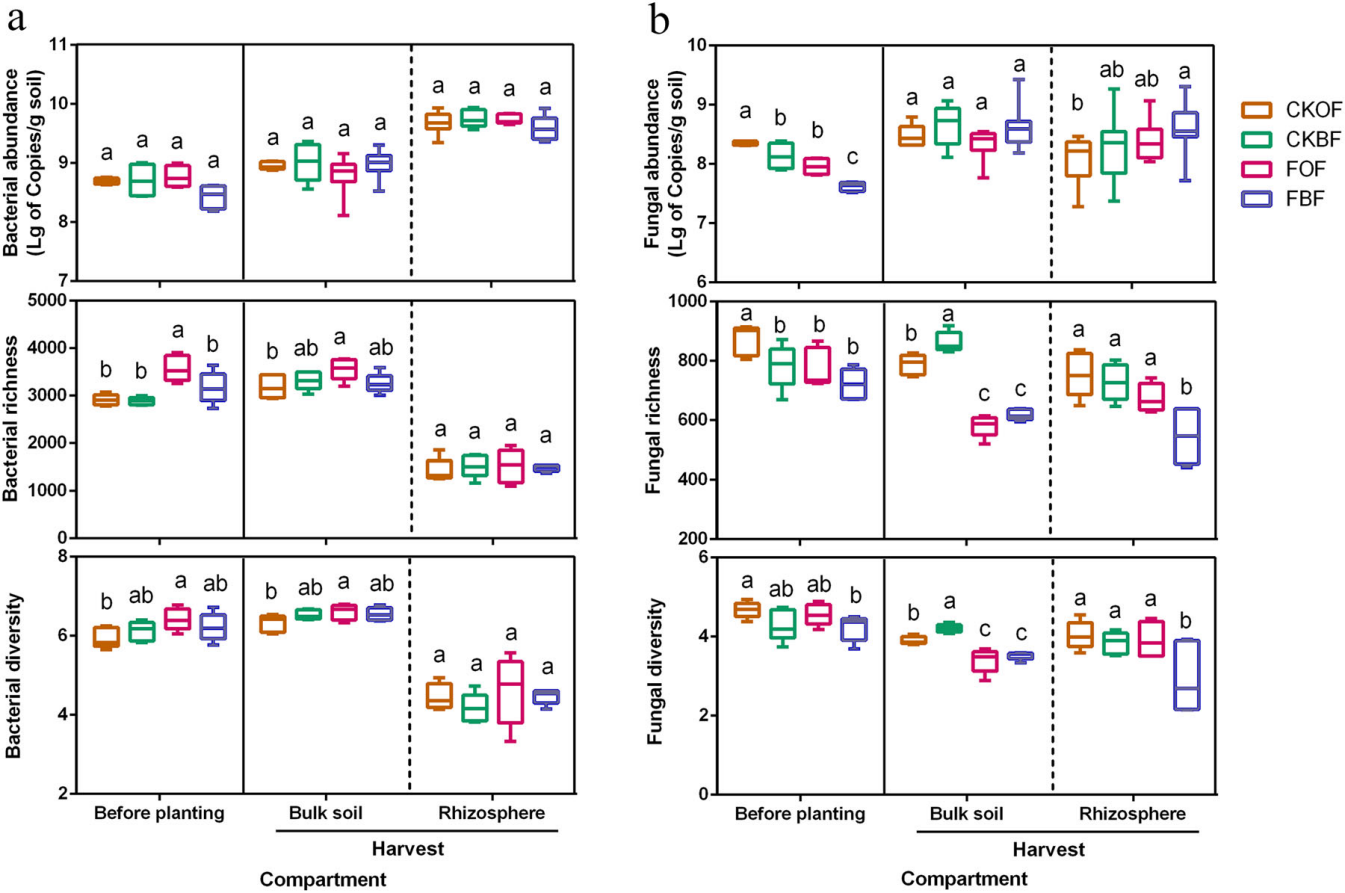
Boxplot of abundance, richness (sobs), and diversity (Shannon) index. **a** bacterial community; **b** fungal community. CKOF: organic fertilizer was amended in un-fumigated soil; CKBF: bio-organic fertilizer was amended in un-fumigated soil; FOF: organic fertilizer was amended in fumigated soil; FBF: bio-organic fertilizer was amended in fumigated soil. Different letters indicate significant differences among the four treatments as defined by Tukey’s test (*p* < 0.05).

For bacteria, although significantly higher bacterial richness (observed number of species) and diversity (Shannon) were observed in FOF in bulk soil before planting and during harvest, no significant value was observed for bacterial abundance, richness, and diversity during harvest in rhizosphere samples (Fig. 3a). For fungi, reductions in fungal abundance, richness, and diversity were detected in the fumigated (FOF and FBF) treatments before planting, and in the bulk soil at harvest. However, in the rhizosphere, only FBF showed significantly lower richness and diversity than the other treatments (Fig. 3b). The correlation between rhizosphere microbiota and disease incidence revealed that fungal richness (*r* = 0.712, *p* = 0.001) and diversity (*r* = 0.480, *p* = 0.037) were positively correlated with disease incidence, while no significant correlations were observed for fungal abundance (*r* = −0.158, *p* = 0.506), bacterial abundance (*r* = 0.434, *p* = 0.056), bacterial richness (*r* = 0.017, *p* = 0.944) or bacterial diversity (*r* = −0.167, *p* = 0.482) (Supplementary Fig. 4).

### Microbial composition manipulated by fumigation and organic amendment

**Fig. 4:**
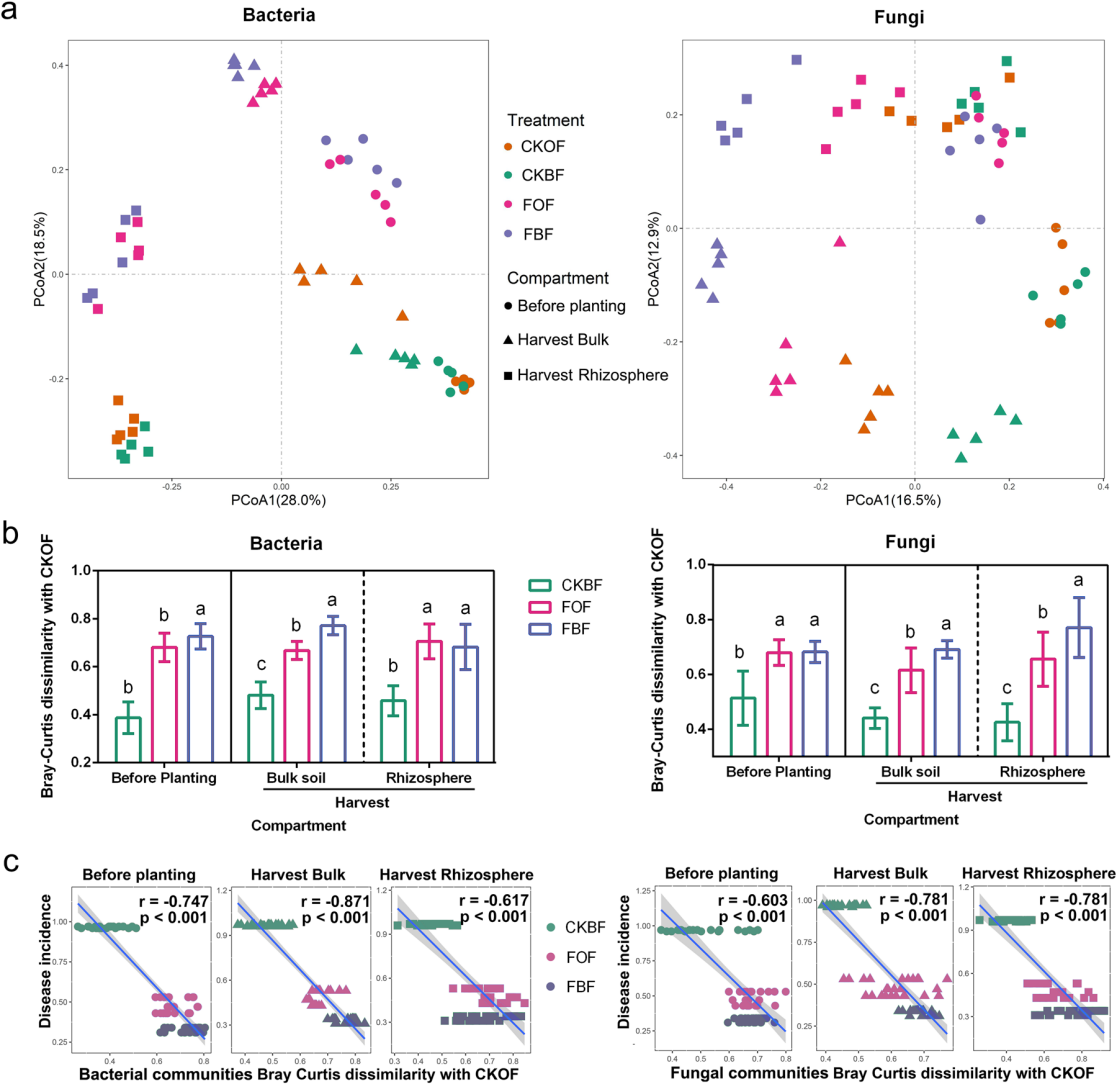
Bacterial and fungal community composition. **a** Principal coordinate analysis (PCoA) ordinations of the bacterial and fungal community composition based on Bray-Curtis distance metric in all soil samples. CKOF: organic fertilizer was amended in un-fumigated soil; CKBF: bio-organic fertilizer was amended in un-fumigated soil; FOF: organic fertilizer was amended in fumigated soil; FBF: bio-organic fertilizer was amended in fumigated soil. Circles refer to the samples before planting, triangles refer to the samples of bulk soil at harvest, and squares refer to the rhizosphere samples at harvest. **b** Bray-Curtis dissimilarity of bacterial and fungal communities between CKOF and other treatments. Different letters indicate significant differences among the four treatments as defined by Tukey’s test (*p* < 0.05). **c** Linear regression between Bray-Curtis dissimilarity in relation to the control treatment and disease incidence. *r* and *p* value were calculated through spearman correlation.

The taxonomic composition of bacterial and fungal communities was similar within fumigated or un-fumigated soil (Supplementary Fig. 5). To visualize the differences in the community composition, principal coordinate analysis (PCoA) based on the Bray-Curtis distances was conducted. The overall bacterial community composition was distinctly separated from that before planting and each compartment of harvest along the first component (PCoA1). In each composition, the bacterial community was distinctly separated between the fumigated and un-fumigated treatments along the second component (PCoA2). For fungi, four treatments were directly separated along the first component (PCoA1) and community composition from bulk and rhizosphere was separated along the second component (PCoA2) during harvest. Moreover, fungal community composition before planting was separated from the fumigated and un-fumigated treatments along the first component (PCoA1) (Fig. 4a, Supplementary Table 3). The bacterial community of CKBF was more similar to that of CKOF than that of FOF and FBF before planting and this phenomenon was also observed in bulk soil and rhizosphere during harvest (Fig. 4b). Moreover, the turnover in bacterial and fungal communities was higher in the fumigated treatment and correlated with disease incidence (*p* < 0.001, Fig. 4c). Furthermore, multiple regression tree (MRT) and variation partitioning analysis (VPA) revealed stronger clustering of the established microbial communities according to fumigation treatment rather than organic amendment. Compared to the influence of fertilizer on the bacterial and fungal communities, fumigation showed more influence in shaping bacterial than fungal composition, in both bulk and rhizosphere soil samples during harvest (Supplementary Fig. 6). The use of other multivariate approaches, such as NMDS, showed similar results (Supplementary Fig. 7), confirming the robustness of the data.

### Relationships among microbial communities, two kinds of pathogens, and plant disease

**Fig. 5:**
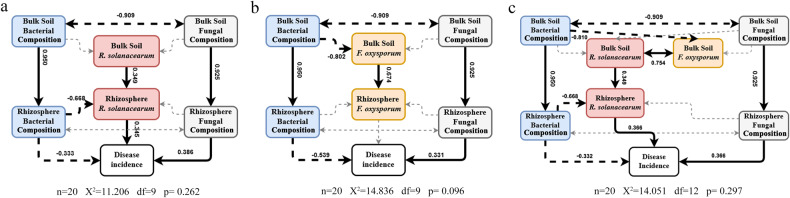
Structure equation model (SEM) of the direct and indirect pathways influencing disease incidence caused by two kinds of pathogens. **a** Only *R. solanacearum* was considered in SEM; **b** only *F. oxysporum* was considered in SEM; **c** both *R. solanacearum* and *F. oxysporum* were considered in SEM (**c**). Arrows represent the flow of causality. Solid and dotted lines represent statistically significant (*P* ≤ 0.05) and non-significant relationships, respectively. The path coefficients associated with each arrow of significant relationships are shown.

Three structural equation models (SEMs) linking shifts in bulk soil and rhizosphere microbial community, pathogens and disease incidence showed that *R. solanacearum* was the better indicator directly causing tomato disease than *F. oxysporum* and the bacterial community was the main indicator suppressing both pathogens (Fig. 5a, b). After a series of failed and successful models were built to combine the two pathogens (Supplementary Fig. 8), a valid model was established to illustrate the relationship between *R. solanacearum* and *F. oxysporum* (Fig. 5c). From the model, tomato wilt disease may be induced by two pathogens, but *R. solanacearum* in the rhizosphere explained more of the disease, whose abundance was suppressed directly by the rhizosphere bacterial community and indirectly by the bulk soil bacterial community through *F. oxysporum* suppression in bulk soil. Hence, the SEM model suggested that the disease was controlled by pathogen decrease via a complex joint effect.

### Potential key taxa

Given the importance of suppressing bulk soil *F. oxysporum* and rhizosphere *R. solanacearum*, the bulk soil and rhizosphere bacterial community composition were further investigated. Taxa with a relative abundance higher than 1% were selected and subjected to Spearman correlation, leading to the identification of three bulk and six rhizosphere taxa. The three sensitive bacterial taxa present in the bulk soil showing a significant negative correlation with *F. oxysporum* abundance were: OTU_6643 (*Ohtaekwangia*; *r* = −0.815, *p* < 0.001), OTU_582 (*Ohtaekwangia*; *r* = −0.820, *p* < 0.001), and OTU_3318 (*Chitinophaga*; *r* = −0.700, *p* = 0.001) (Table 2). The six rhizosphere-sensitive bacterial taxa showing a significant negative correlation with *R. solanacearum* were identified as OTU_8463 (*Chitinophaga*; *r* = −0.681, *p* = 0.001), OTU_346 (*Chitinophaga*; *r* = −0.592, *p* = 0.006), OTU_1868 (Olivibacter; *r* = −0.670, *p* = 0.001), OTU_8641 (*Flavihumibacter*; *r* = −0.641, *p* = 0.002), OTU_6257 (*Flavobacterium*; *r* = −0.598, *p* = 0.005), and OTU_3519 (*Terrimonas*; *r* = −0.668, *p* = 0.001) (Table 2). Moreover, the co-occurrence analysis also showed that *Ohtaekwangia* was negatively correlated with *F. oxysporum* in bulk soil, whereas *Chitinophaga* and *Olivibacter* revealed a negative correlation with *R. solanacearum* in the rhizosphere (Supplementary Fig. 9).

**Table 2:** Spearman correlation between bacterial OTUs and pathogens.

Compartment	Taxa	Genus	Relative abundance in each treatments				Spearman correlation	
			FBF	FOF	CKOF	CKBF	*r*	*p*
Bulk soil (with *F. oxysporum*)	OTU_6643	*Ohtaekwangia*	2.26%	3.03%	0.07%	0.02%	−0.815	<0.001
	OTU_582	*Ohtaekwangia*	1.57%	1.24%	0.09%	0.00%	−0.820	<0.001
	OTU_3318	*Chitinophaga*	1.50%	0.66%	0.11%	0.03%	−0.700	0.001
Rhizosphere (with *R. solanacearum*)	OTU_8463	*Chitinophaga*	8.93%	11.43%	1.09%	0.10%	−0.681	0.001
	OTU_346	*Chitinophaga*	4.08%	3.43%	1.04%	0.03%	−0.592	0.006
	OTU_1868	*Olivibacter*	1.63%	3.37%	0.25%	0.10%	−0.670	0.001
	OTU_8641	*Flavihumibacter*	2.03%	1.83%	0.53%	0.02%	−0.641	0.002
	OTU_6257	*Flavobacterium*	0.56%	2.47%	0.04%	0.00%	−0.598	0.005
	OTU_3519	*Terrimonas*	1.25%	1.44%	0.08%	0.01%	−0.668	0.001

Only the OTUs (relative abundance >1%) with significant (*p* < 0.05) and negative correlation with pathogens abundance were showed.

## Discussion

To reduce the environmental footprint of agriculture, there is an urgent need to develop practices that improve soil resistance or resilience to disease, and therefore reduce the use of pesticides while maintaining high agricultural productivity. Here, we described two strategies—fumigation and organic amendment—that can potentially lead to soil suppression against multi-pathogen system. Our results revealed that when the addition of bio-organic fertilizer is preceded by an initial disturbance of the soil microbiome through fumigation, it is possible to control tomato wilt disease and suppress *R. solanacearum* and *F. oxysporum* effectively. Below we discuss these results in light of specific changes in microbiome composition by deciphering the mechanism involved in the multi-pathogen suppression.

**Fig. 6:**
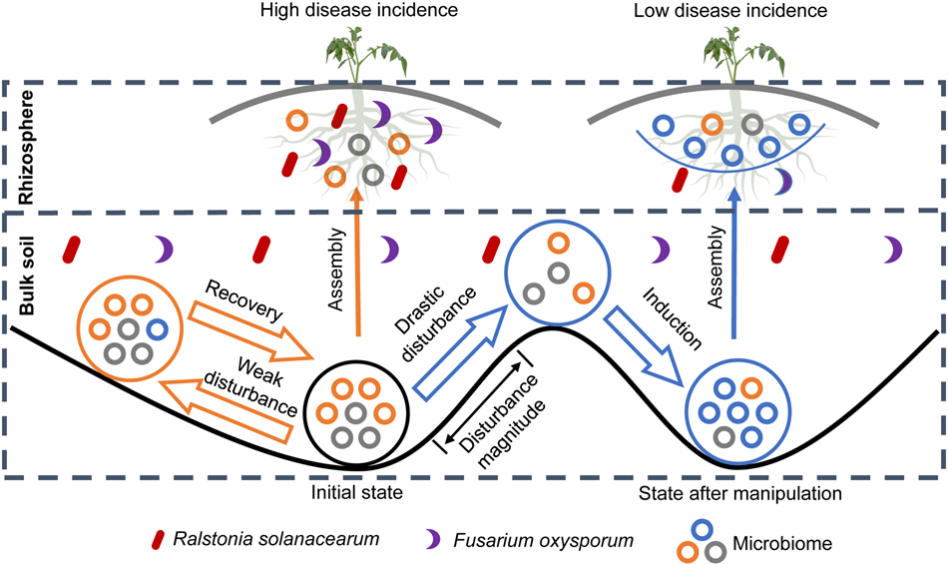
Main mechanism of soil manipulation targeting multi-pathogen suppression. Orange arrows showed the influence of weak disturbance (bio-organic amendment) on initial microbiome, whereas blue arrows showed the influence of strong disturbance (fumigation) on initial microbiome. When weak disturbance is applied, the microbial community is either resistant or highly resilient to the changes, subsequently returning to initial diseased state. While after fumigation, the initial state of microbiome is drastically perturbed, ensuring the development of an alternative community composition that develops into a healthy soil microbiome in response to organic amendments, achieving low disease incidence in subsequent plant growth.

Soil microbial communities are often expected to be resilient to perturbations given their high taxonomic diversity and functional redundancy^31–33^. However, certain disturbances, such as those caused by fumigation, are known to exert drastic effects on both the taxonomic and functional components of the soil microbiome communities through ammonia stress and shifts in soil^8,34^. In our study, we showed that fumigation indeed induced high turnover in both bacterial and fungal composition, indicating that fumigation regulates the variation in microbial composition^10^. Conversely, fertilization with either organic or bio-organic fertilizer represented a weak disturbance to which microbial communities were resistant (Fig. 6). Whereas the latter disturbance is inefficient from a disease control perspective, even in the presence of biocontrol agents, fumigation might be too severe, disrupting microbial interactions along with the pathogens, with unpredictable long-term effects.

Ecological theory predicts that initial disturbances affect the stability of soil microorganisms to subsequent disturbances^4^, indicating that compounded perturbations can strongly change the microbial structure, especially when communities experience divergent stresses^35^. Previous studies have observed that fumigation disturbance alone can retain some potential beneficial microbes^36^, while in this study, we used a similar principle by applying a strong disturbance in the form of fumigation, which paved the way for the beneficial microorganisms and organic amendments present in the fertilizers, thus prompting an alternative state in community composition. Importantly, these taxonomic changes had functional consequences for disease suppression regardless of the type of organic fertilizer used.

Organic amendments are known for their ability to alleviate environmental stress^37^, encourage the growth and activity of soil microbial populations^38^ and induce specific taxa in the soil microbial community^39^, broadly influencing the soil microbiome^12,29^. Although both fertilizers led to suppressiveness, we still observed variations in microbial composition, especially for fungi, indicating that the degree of suppressiveness might be a redundant function.

Although the approach proposed here might sound extreme given that previous chemical fumigants were harmful to high-value agricultural production systems because of their high toxicity to the environment and nearby residents^40^, the new generations of fumigants used in this study are considered more environmentally friendly. For instance, several reports suggest that high-N amendments can promote suppression of pathogens and root-knot nematodes^41,42^, which indicates a potential positive effect of the ammonia released by the application of ammonium bicarbonate. Furthermore, calcium oxide is a widely used soil remediation agent to remediate heavily contaminated soil and allow revegetation^43^. Finally, we showed that the subsequent application of organic amendment counteracted any potential negative effects associated with fumigation, by improving microbial activity and influencing the diversity and structure of the microbial communities. Thus, we assumed that this novel approach, which combines a less harmful fumigant with an organic amendment, is environmentally friendly.

Previous literature has reported that fumigation using ammonium bicarbonate and calcium oxide showed strong antifungal effects by releasing ammonium to destroy the hyphae of fungal pathogens^8^. Before planting, the abundance of *F. oxysporum* in soil decreased with fumigation, and this effect was maintained until the harvest period, even in the rhizosphere soil, a result that was also found in cucumber^44^ and banana^10^ diseased soil in response to fumigation. Thus, fumigation decreased the pathogen load, leading to direct suppression.

While *F. oxysporum* maintained a relatively stable low abundance during tomato growth in this study, we also expect microbes to indirectly contribute to the suppression of this pathogen^45^. At the genus level, higher abundances of *Ohtaekwangia* and *Chitinophaga* were observed in the two fumigation treatments (Supplementary Fig. 10) in bulk soil, with a significant difference between the fumigated and un-fumigated treatments during harvest and before planting. Thus, the negative relationships between *F. oxysporum* and *Ohtaekwangia* or *Chitinophaga* suggest that these microbes may represent key taxa involved in fungal pathogen suppression. *Ohtaekwangia* has been reported to produce bioactivity against *Plasmodium falciparum*^46^, whereas *Chitinophaga* can exhibit fungicidal activities and induce antagonistic traits in other bacterial taxa^47^. We, therefore, speculate that the increase in the abundance of these species represents a follow-up effect of the two-step strategy. Overall, we speculate that fungal pathogens were suppressed by fumigation directly, via hyphal destruction, and indirectly, through the effect of fumigation on bacterial community assembly.

Unlike the performance of *F. oxysporum*, fumigation did not reduce the number of *R. solanacearum* at the time before planting. One report showed that ammonium could only reduce the growth of *R. solanacearum* at pH 9^48^, and the soil pH in this study was less than 7. Although the abundance of *R. solanacearum* increased with tomato planting, significantly lower values were observed for fumigated treatments in bulk and rhizosphere soil during harvest compared to un-fumigated soil, suggesting that fumigated soil possessed the ability to suppress the pathogen. The soil type of this study did not show a significant correlation with pathogens (Table 1). In accordance with our results, many reports have shown that the soil microbiome contributed the pathogen suppression based on soil disinfection^45,49^, which indicate that soil properties could not directly suppress pathogens. Thus, we speculate that the soil microbiome contributed to the suppression of bacterial pathogen.

The soil bacterial diversity and composition are often positively correlated with pathogen suppression^50,51^. In this study, bacterial composition was correlated with the suppression of bacterial pathogens (Fig. 5a), confirming previous findings in suppressive soil systems induced by bio-organic fertilizer and organic amendment^52,53^. Thus, we deduced that fumigation suppressed bacterial pathogens indirectly through shifts in bacterial community composition. At the genus level, higher abundances of *Chitinophaga*, *Olivibacter*, *Flavihumibacter*, *Flavobacterium*, and *Terrimonas* were observed in the rhizosphere soil samples from fumigation treatments (Supplementary Fig. 11). *Chitinophaga* can produce potentially resistant substances, such as elansolid^54^, to suppress pathogenic bacteria. *Flavihumibacter*, belonging to *Chitinophagaceae*, also has the potential to suppress pathogens^55^. Moreover, *Flavobacterium* was found to suppress *R. solanacearum* in pot experiments^56^, whereas *Terrimonas* exhibited a negative relationship with apple replant disease^57^ and showed a greater abundance in the rhizosphere of healthy *Panax notoginseng*^58^. However, there is no evidence in the literature for the potential role of *Olivibacter* in suppressing pathogens. Overall, the negative relationships between *Chitinophaga*, *Flavihumibacter*, *Flavobacterium*, and *Terrimonas* in the rhizosphere suggest that these microbes are potential key taxa involved in bacterial pathogen suppression. Although most of these species were found in higher abundance in the bulk soil of the CKOF treatment during harvest, all species showed consistently significant differences between the fumigated and un-fumigated treatments in the rhizosphere. We, therefore, speculate that these species mainly represent an effect of the two-step strategy.

Our results showed that *R. solanacearum* and *F. oxysporum* undertake several interactions in soil (Fig. 5, Supplementary Fig. 12). *R. solanacearum* is known to persist in soils for a remarkably long period of time relative to other bacterial plant pathogens^59^, which has been postulated to be controlled by soil fungi (*Fusarium*, *Aspergillus* et al.)^60^. This highlights the importance of considering complex pathogen interactions when designing new strategies to improve the growth and health of crops^61,62^. Importantly, the fumigation + organic amendment strategy used here not only reduced the damage caused by each bacterial and fungal pathogen but also potentially decreased the disease outcome by eliminating any potential synergic interactions between the two pathogens.

The two-step strategy proposed in this study provides a robust concept to manipulate the microbiome to improve plant health. Based on the literature and our research, we assume that for agricultural ecosystem management, when organic fertilizer (weak disturbance) is directly applied to the soil microbiome, the strength of the disturbance is not sufficient to induce functional shifts from conducive to suppressive soils. However, when preceded by a strong disturbance in the form of fumigation, the soil microbiome was greatly affected—fungal pathogens were directly suppressed, whereas the assembly of bacteria and fungi was further redirected by organic amendments towards suppressive communities (Fig. 6).

In summary, the results of this study confirmed that fumigation combined with organic amendment represents an effective strategy for controlling tomato diseases caused by multiple pathogens. The strategy directly decreased fungal pathogens by fumigation but indirectly suppressed both fungal and bacterial pathogens through changes in bacterial but not fungal communities. Specifically, the beneficial microbiome was induced by drastic disturbance caused by fumigation, stimulating suppression in an indirect fashion. The mechanism of multi-pathogen suppression remains a subject for future study to elucidate its role in disease outcome and permit the design of better agricultural management and its use in sustainable strategies for plant health.

## Methods

### Field description

A field experiment was performed in the town of Hengxi in Nanjing, Jiangsu Province (32°02′N, 118°50′E), which has a tropical monsoon climate with an average annual temperature and precipitation of 15.4 °C and 1106 mm, respectively. The experiment was continuously performed for three field seasons, from March 2014 to June 2015, with two seasons in 2014 and one in 2015. The field experiment consisted of 4 treatments: CKOF, 0.3 kg/m^2^ organic fertilizer (OF) was amended in un-fumigated (CK) soil; CKBF, 0.3 kg/m^2^ bio-organic fertilizer (BF) was amended in un-fumigated soil; FOF, 0.3 kg/m^2^ organic fertilizer was amended in fumigated (F) soil; and FBF, 0.3 kg/m^2^ bio-organic fertilizer was amended in fumigated soil. Each treatment had three randomized independent replicate plots and each replicate plot contained 40 tomato plants (Supplementary Fig. 1). The tomato variety used in these experiments was “Shi Ji Fen Guan”, which is one of the early ripening tomato cultivars with pink, round, large fruit, that is normally cropped in this area and susceptible to soil-borne disease^20^. Fumigation was achieved by mixing 0.15 kg/m^2^ ammonium bicarbonate and 0.15 kg/m^2^ lime, and after application of fumigant, all treatments were covered with plastic film for 15 days before fertilization. Fertilization consisted of applying chicken manure compost (N: 2.0%, P: 0.9%, K: 0.9%) or bio-organic fertilizer (N: 2.2%, P: 1.0%, K: 1.0%) to treatments OF and BF, respectively. We compensated for the nutrient differences between the two types of fertilizers with mineral fertilizer. Bio-organic fertilizer was produced by inoculation of *Bacillus amyloliquefaciens* T-5^63^ into an organic mixture of rapeseed meal and chicken manure composts at a ratio of 1:4 (dw/dw) for the solid fermentation process.

### Assay of tomato disease incidence

In each season, disease incidence was recorded when most of the tomato fruits were ripe. The symptoms caused by *R. solanacearum* are wilted leaves that maintain a green color, and the vascular tissues in the lower stem of wilted plants show dark brown discoloration. The symptoms caused by *F. oxysporum* f. sp. *Lycopersici* were older leaves that drooped, curved downward, and turned yellow, and the vascular tissue of a diseased plant was dark brown^64^. In this study, diseased tomato showed bacterial and Fusarium wilt symptoms simultaneously. Therefore, we defined diseased plants based on observations of typical wilt symptoms^10,65^, for instance, necrosis and drooping of the leaves. The Disease incidence of the field experiment was calculated by counting the number of tomato plants with wilt symptoms among the total number of plants, and each treatment in each season had three replicates. To minimize the effect of personal observation, the diseased plants were counted twice by two persons, separately.

### Soil sampling, preparation, and soil chemical analysis

Soil samples were collected before planting and during harvest in 2015. Samples collected on the 7th day after fumigation before fertilization in March were defined as before planting because tomato seedlings were transplanted after fertilization, and bulk and rhizosphere soil samples collected during harvest in June were defined as harvesting period samples. For bulk soil sample collection before planting, in brief, a nine-point sampling method was utilized to collect soil cores (diameter is 50 mm) with 0–15 cm depth from the surface in each plot to form a composite sample, and we performed two nine-point samplings in each plot to form two composite samples as two replicates so that we had six bulk soil replicates for each treatment. For bulk soil sample collection at harvest, we collected nine soil cores with 0–15 cm depth from the surface after removing plants in each plot to form a composite sample as one replicate, and we sampled two replicates in each plot as well as before planting. All bulk soil samples in each replicate were subsequently mixed individually in a 2-mm sieve to homogenize the soil, one portion of each sample was stored at −80 °C for further DNA extraction, and the other portion was air-dried for chemical analyses. All bulk soil chemical properties were determined according to Liu et al.^20^. For rhizosphere samples, two tomato roots were collected from each plot and shaken vigorously to remove excess soil, and then the soil adhering to the roots (rhizosphere soil) was removed by sterile water that was obtained through 30 min autoclave in 121 °C. We collected two rhizosphere samples from each plot, which were frozen and stored at −80 °C for soil DNA extraction. Thus, 6 bulk and 6 rhizosphere soil samples were collected for each treatment.

### DNA extraction and quantitative PCR determination

Five bulk and rhizospheric soil samples from each treatment and period were randomly chosen for subsequent DNA extraction, and 0.25 g soil of each sample was used for the DNA extraction using the Power Soil DNA Isolation Kit (MoBio Laboratories Inc., USA) following the manufacturer’s protocol. Then the DNA samples were measured through a spectrophotometer (NanoDrop 2000, USA) to ensure that they were available for amplicon sequencing. The abundance of bacteria (338F and 518R), fungi (ITS1F and 5.8S), *Ralstonia solanacearum* and *Fusarium oxysporum* f. sp. *Lycopersici* were quantified by quantitative PCR (qPCR) with primers described in Supplementary Table 1. The qPCR analyses were carried out with an Applied Biosystems 7500 real-time PCR system (Applied Biosystems, CA) using SYBR green I fluorescent dye detection in 20-μl volumes containing 10 μl of SYBR Premix Ex Taq (TaKaRa Bio Inc., Japan), 2 μl of template, and 0.4 μl of both forward and reverse primers (10 mM each). All qPCR reactions were performed using the standard temperature profile^66^. Each sample was analyzed in three replicates to remove systematic error, and the results are expressed as log10 values (target copy number g^−1^ soil).

### Sequencing of bacterial and fungal ribosomal markers

The bacterial 16S rRNA gene V4 region was amplified from soil genomic DNA by primers 520F and 802R, while ITS1F and ITS2 were used for amplification of the fungal internal transcribed spacer 1 (ITS1) region (primers are described in Supplementary Table 1). Amplicons were sequenced using the Illumina MiSeq PE250 platform at Personal Biotechnology Co., Ltd, Shanghai, China.

Samples were analyzed using the UPARSE pipeline^67^. All the scripts used in this study to run the UPARSE pipeline are available in the Supplementary material. Briefly, forward and reverse reads, directly obtained from the company, were merged using the fastq_mergepairs command in USEARCH v10.0. Quality control was carried out using the fastq_filter command in USEARCH. The unique sequence reads were obtained with the de-replication and de-singleton command fastx_uniques, high-quality sequences were subsequently clustered into operational taxonomic units (OTUs, 97% similarity) with USEARCH. This step generated a 16S rRNA gene OTU table of 60 samples × 9163 OTUs (6,390,357 reads) and an ITS1 gene table of 60 samples×2955 OTUs (5,061,532 reads). The number of high-quality sequences per sample varied from 42,254 to 169,009 for bacteria and 14,364 to 125,125 for fungi. Finally, classification of the representative sequences for each OTU was performed using the RDP classifier for bacteria 16S rRNA gene and the UNITE database for fungi ITS1 gene^68^. After classification, combined with the NCBI database, the untargeted organisms (such as archaea, chloroplasts, and mitochondria) were identified and removed. To obtain an equivalent sequencing depth for further bacterial and fungal community analysis, each sample was rarefied to 42,128 sequences for 16S rRNA genes and 14,364 sequences for ITS sequences in MOTHUR^69^. Moreover, after rarefaction, the average Good’s coverage of the remaining 16S and ITS sequences left was 97.3 ± 0.7% and 99.3 ± 0.2%, respectively (Supplementary Table 2).

### Statistical analysis

All statistical tests performed in this study were considered significant at *p* < 0.05. First, microbial community richness (Sobs) and evenness (Shannon) were calculated in MOTHUR. One-way analysis of variance (ANOVA) among treatments of each compartment was used to examine the effect of the two-step strategies. The liner regression and Spearman correlations between microbial indexes and disease incidence were analyzed to reveal their potential contribution to disease suppression. Then, we performed principal coordinate analysis (PCoA) and non-metric multidimensional scaling (NMDS) based on Bray-Curtis distance to compare the major similarity and variance components of the bacterial and fungal community compositions among all soil samples. To support the PCoA result, analysis of molecular variance (AMOVA) was calculated to evaluate the significant differences in bacterial and fungal community structures among the four treatments in MOTHUR. We also constructed multivariate regression trees (MRT) and variation partitioning analysis (VPA) to estimate explanatory variables contributing to community differences. Several structural equation models (SEMs) were used to explore the mechanism of multi-pathogen suppression. Finally, because SEMs pointed that bulk bacteria suppressed *F. oxysporum* and rhizosphere bacteria suppressed *R. solanacearum*, the Spearman correlation between the relative abundance of bulk OTUs and abundance of bulk *F. oxysporum* was used to indicate potential suppressive OTUs in bulk soil, and the Spearman correlation between the relative abundance of rhizosphere OTUs and abundance of rhizosphere *R. solanacearum* was used to indicate potential suppressive OTUs in rhizosphere. PCoA, NMDS, MRT, VPA, SEM, and linear regression were calculated through R version 3.4.0 for Windows. The respective scripts can be found in the Supplementary material. For other statistical analyses, Spearman correlations, ANOVA, Tukey’s test, and two-sample *t*-test analyses were calculated in IBM SPSS 23.0.

### Reporting summary

Further information on research design is available in the Nature Research Reporting Summary linked to this article.

## Supplementary information

Supplementary Information

Reporting Summary

## Data Availability

The raw sequence data for the 16S rRNA gene V4 region and the ITS1 region of all samples were submitted to the NCBI Sequence Read Archive database (https://www.ncbi.nlm.nih.gov/) with the accession number SRP188824 (PRJNA527805).

## Code availability

The scripts can be found in the Supplementary material.
