# Supplementary material for: Soil microbiome manipulation triggers direct and possible indirect suppression against Ralstonia solanacearum and Fusarium oxysporum
Source: NPJ Biofilms Microbiomes. 2021 Apr 12;7:33. doi: 10.1038/s41522-021-00204-9 (PMC8041757; doi:10.1038/s41522-021-00204-9)
Supplement: Supplementary file 1 — Supplementary Information [file 41522_2021_204_MOESM1_ESM.pdf]

Article title: Soil microbiome manipulation triggers direct and possible indirect suppression against *Ralstonia solanacearum* and *Fusarium oxysporum*

Authors: Xuhui Deng, Na Zhang, Zongzhuan Shen, Chengzhi Zhu, Hongjun Liu, Zhihui Xu, Rong Li, Qirong Shen, Joana Falcao Salles

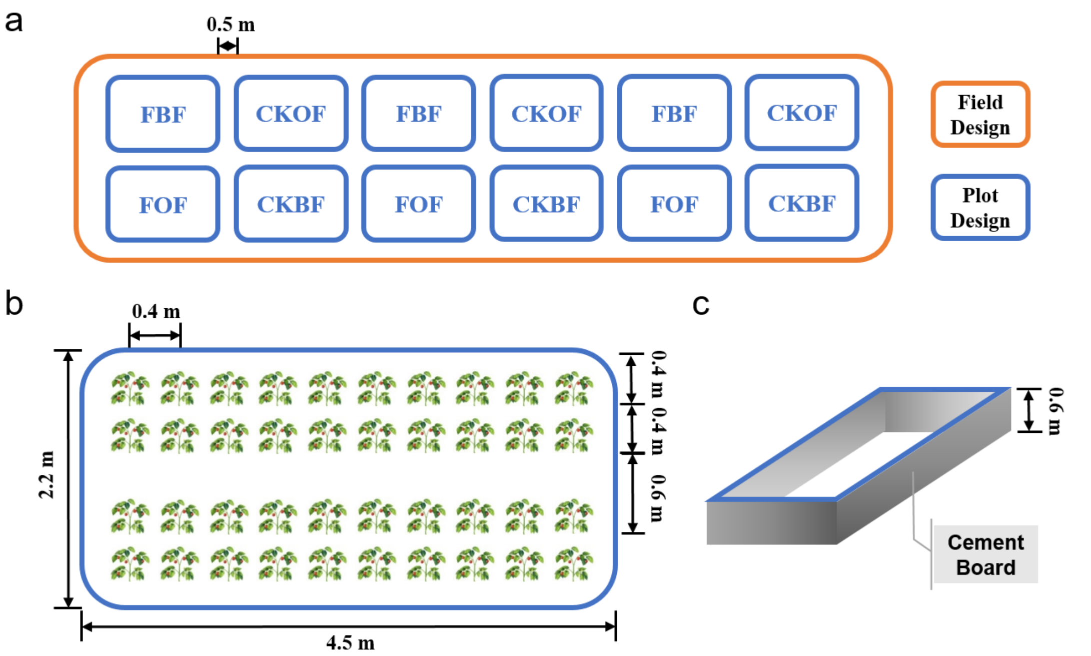

Supplementary Fig 1 field experiment design. a. plots distribution of the field experiment. b. plants distribution of each plot. c. the separation method among plots. CKOF: organic fertilizer was amended in un-fumigated soil; CKBF: bio-organic fertilizer was amended in un-fumigated soil; FOF: organic fertilizer was amended in fumigated soil; FBF: bio-organic fertilizer was amended in fumigated soil.

Supplementary Table 1 Sequences of oligonucleotide primers required for quantitative PCR

Target group	Primer name and sequence (5´-3´)	Reference
Bacteria	338F: ACTCCTACGGGAGGCAGCAG	[1]
	518R: ATTACCGCGGCTGCTGG	
Fungi	ITS1F: TCCGTAGGTGAACCTGCGG	[1]
	5.8S: CGCTGCGTTCTTCATCG	
R.. solanacearum	FliCF: GAACGCCAACGGTGCGAACT	[2]
	FliCR: GGCGGCCTTCAGGGAGGTC	
F. oxysporum f. sp. *Lycopersici*	sp1-2f: GCTGGCGGATCTGACACTGT	[3]
	sp1-2r: TTTCGTACTTGCCAGGTTG	
Bacterial 16S RNA gene V4	520F: AYTGGGYDTAAAGNG	[4]
	802R: TAC NVG GGT ATC TAA TCC	
Fungal ITS1	ITS1F: CTTGGTCATTTAGAGGAAGTAA	[5]
	ITS2: GCTGCGTTCTTCATCGATGC	[6]

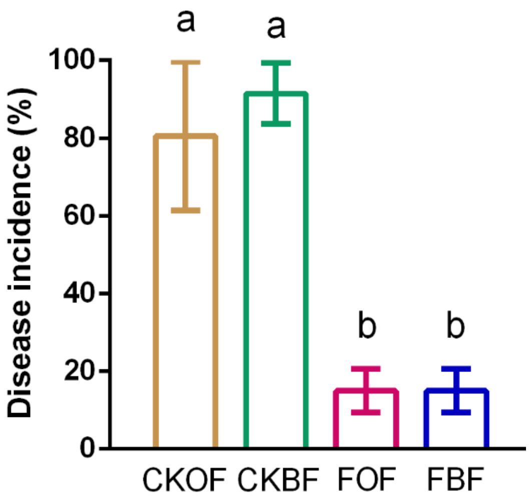

Supplementary Fig 2 Results of tomato wilt disease incidence in Jun, 2014. CKOF: organic fertilizer was amended in non-fumigated soil; CKBF: bio-organic fertilizer was amended in non-fumigated soil; FOF: organic fertilizer was amended in fumigated soil; FBF: bio-organic fertilizer was amended in fumigated soil. All values are the mean of three replicates. Bars with different letters indicate significant differences among the four treatments as defined by Tukey's test ($p<0.05$)

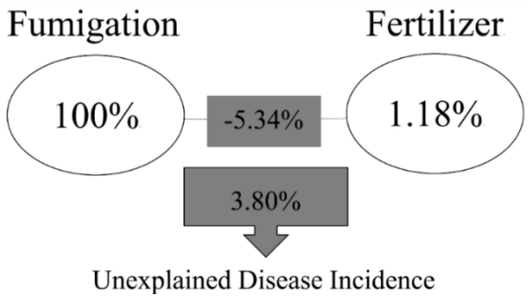

Supplementary Fig 3 variation partitioning analysis (VPA) map of the effects of fumigation and fertilizer type, and interactions of these factors on the disease incidence.

Supplementary Table 2 The number of passing sequences after quality control and Good's coverage values for both bacteria and fungi

Compartment	Sample ID	Passing sequences		Good's coverage	
		Bacteria	Fungi	Bacteria	Fungi
Before Planting	CKOF1	105944	67255	0.986	0.996
	CKOF2	91460	51874	0.985	0.995
	CKOF3	63802	89781	0.986	0.995
	CKOF4	91281	87444	0.987	0.996
	CKOF5	76806	95735	0.986	0.995
	CKBF1	97331	103788	0.982	0.995
	CKBF2	79766	125125	0.984	0.995
	CKBF3	83556	76055	0.986	0.995
	CKBF4	62977	14364	0.989	0.995
	CKBF5	96836	91789	0.990	0.996
	FOF1	94167	95912	0.987	0.993
	FOF2	77396	71338	0.982	0.994
	FOF3	112934	103866	0.990	0.994
	FOF4	83801	120361	0.988	0.994
	FOF5	98664	116674	0.986	0.995
	FBF1	68891	83806	0.990	0.995
	FBF2	87028	115308	0.984	0.994
	FBF3	82970	94519	0.983	0.994
	FBF4	89378	95774	0.987	0.994
	FBF5	100839	80097	0.985	0.994
Bulk Soil Harvest Time	CKOF1	67477	91527	0.976	0.994
	CKOF2	64319	48046	0.973	0.994
	CKOF3	42254	71216	0.975	0.994
	CKOF4	45547	78436	0.975	0.993
	CKOF5	69976	72973	0.975	0.994
	CKBF1	76151	103593	0.973	0.994
	CKBF2	120992	72272	0.972	0.994
	CKBF3	76715	73577	0.971	0.994
	CKBF4	83396	86274	0.970	0.994
	CKBF5	94504	51708	0.972	0.995
	FOF1	134487	96740	0.976	0.993
	FOF2	113724	120849	0.972	0.992
	FOF3	131978	116760	0.972	0.993
	FOF4	143659	88434	0.974	0.993
	FOF5	122023	120336	0.975	0.993
	FBF1	120261	95035	0.974	0.993
	FBF2	113304	78187	0.973	0.992
	FBF3	158964	78441	0.973	0.993
	FBF4	127628	87161	0.976	0.992
	FBF5	151684	81342	0.973	0.993

	CKOF1	116237	47449	0.972	0.994
	CKOF2	165403	53998	0.974	0.994
	CKOF3	169319	45389	0.977	0.994
	CKOF4	120080	53268	0.976	0.995
	CKOF5	120682	41097	0.977	0.995
	CKBF1	131111	44221	0.970	0.994
Rhizosphere	CKBF2	169009	76643	0.972	0.995
Harvest Time	CKBF3	155318	82091	0.969	0.995
	CKBF4	138453	115606	0.972	0.994
	CKBF5	168553	73504	0.971	0.994
	FOF1	145749	100757	0.977	0.993
	FOF2	136055	79157	0.977	0.993
	FOF3	98694	90176	0.977	0.993
	FOF4	112834	114796	0.977	0.993
	FOF5	145511	87205	0.977	0.994
	FBF1	87097	105922	0.978	0.993
	FBF2	56243	89737	0.979	0.993
	FBF3	105985	66024	0.977	0.994
	FBF4	107429	100360	0.979	0.994
	FBF5	135725	100360	0.976	0.993
	Mean				
	Total	6390357	5061532		

The Good’s coverage values were calculated after rarefaction for each sample.

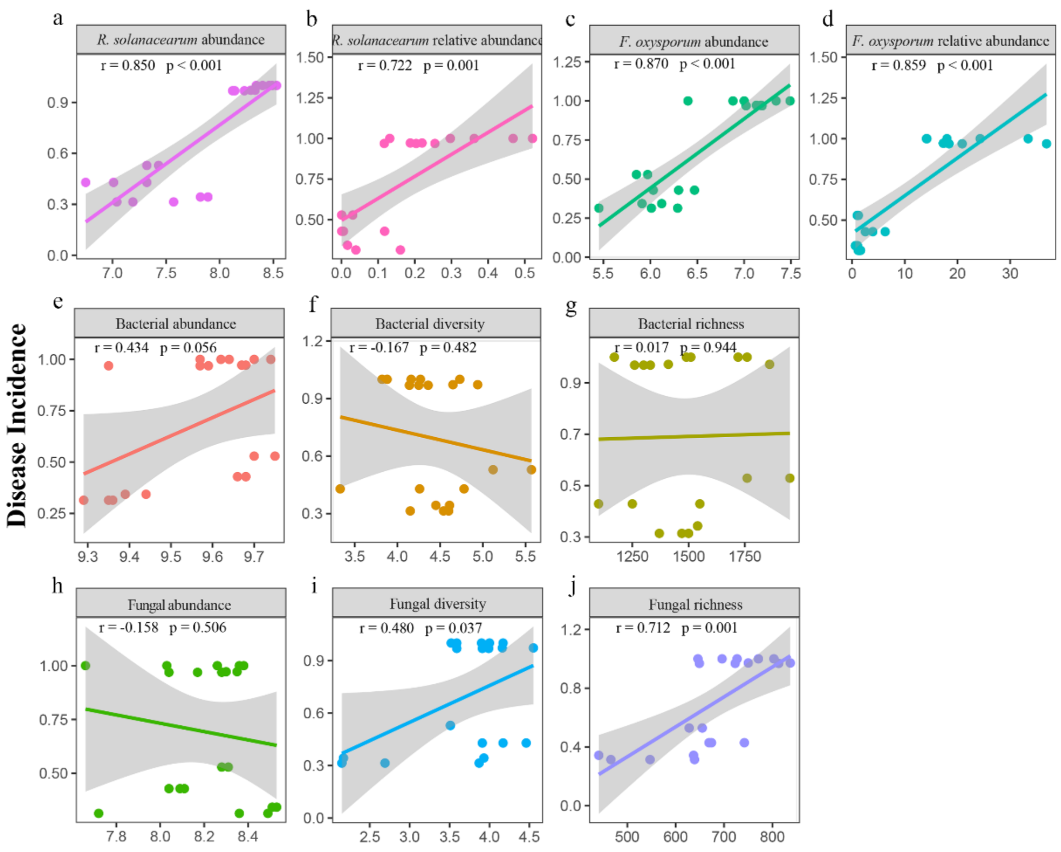

Supplementary Fig 4 Spearman correlations between abundances of *R. solanacearum*, relative abundance of *Ralstonia*, abundances of *F. oxysporum*, relative abundance of *Fusarium*, bacterial abundance (log10 copies per gram soil), bacterial richness (Sobs), bacterial diversity (Shannon), fungal abundance (log10 copies per gram soil), fungal richness (Sobs), fungal diversity (Shannon) and tomato wilt disease incidence.

Supplementary Table 3 Microbial community dissimilarity comparison among treatments using nonparametric statistical method of analysis of molecular variance (AMOVA)

		All treatments	CKOF-CKBF	FOF-FBF
Bacterial	Before planting	**<0.001**	**0.007**	**<0.001**
	Bulk soil	**<0.001**	**0.011**	**0.002**
	rhizoshphere	**<0.001**	**0.030**	**0.024**
Fungi	Before planting	**<0.001**	**<0.001**	**0.007**
	Bulk soil	**<0.001**	**<0.001**	**<0.001**
	rhizoshphere	**<0.001**	0.090	**<0.001**

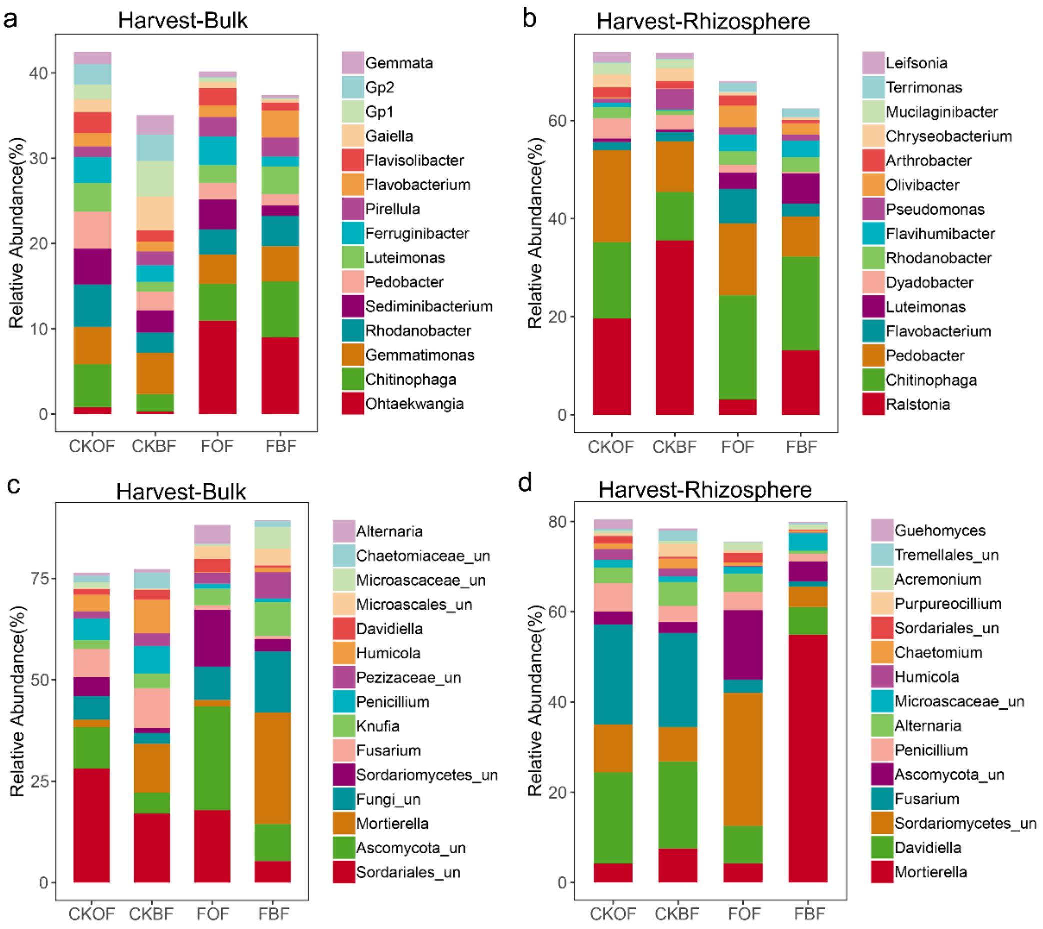

Supplementary Fig 5 Relative abundances of the major Bacterial (a, b) and fungal (c, d) taxa. CKOF: organic fertilizer was amended in un-fumigated soil; CKBF: bio-organic fertilizer was amended in un-fumigated soil; FOF: organic fertilizer was amended in fumigated soil; FBF: bio-organic fertilizer was amended in fumigated soil. All values of each treatment are the mean of five replicates.

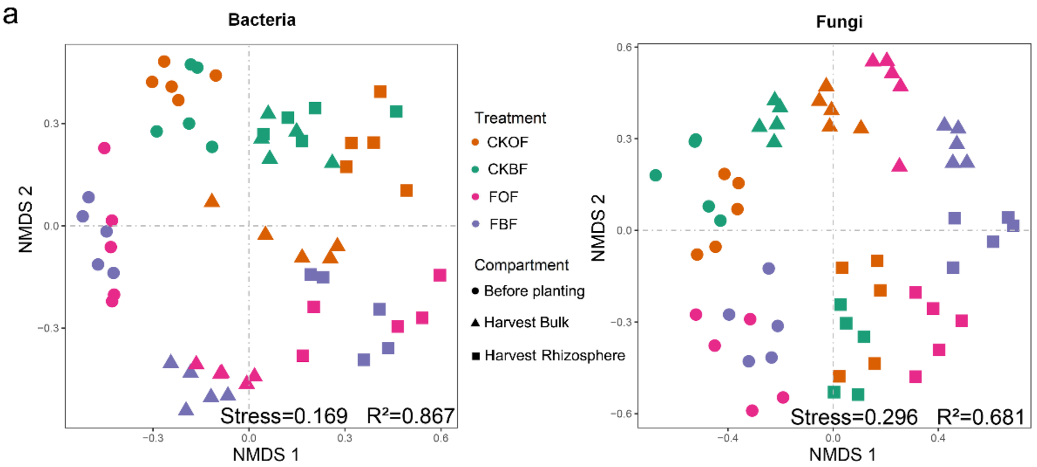

Supplementary Fig 6 Nonmetric multidimensional scaling (NMDS) ordinations of the bacterial and fungal community composition based on Bray-Curtis distance metric in all soil samples. CKOF: organic fertilizer was amended in un-fumigated soil; CKBF: bio-organic fertilizer was amended in un-fumigated soil; FOF: organic fertilizer was amended in fumigated soil; FBF: bio-organic fertilizer was amended in fumigated soil. Circles refer to the samples before planting, triangles refer to the samples of bulk soil at harvest, and squares refer to the rhizosphere samples at harvest.

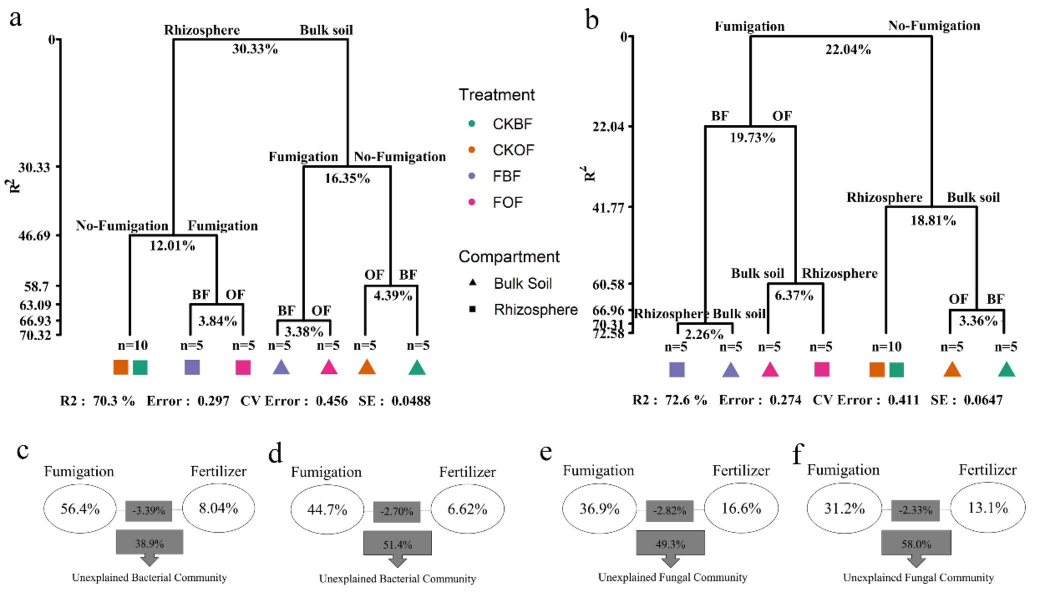

Supplementary Fig 7 Multiple regression tree (MRT) analysis and variation partitioning analysis (VPA) of the microbial community. MRT and VPA all showed treatment effects on the (a) bacterial and (b) fungal community composition. The identity and number of rhizosphere soil samples included in the analysis are shown under the tree. Numbers under the crosses of each split indicate the percentages of variance explained by the split. The R^2, error, cross-validation error (CV Error), and standard error (SE) of the MRT analysis are listed under the tree. c (Bulk soil) and d (Rhizosphere) showed the bacterial composition at harvest, e (Bulk soil) and f (Rhizosphere) showed the fungal composition at harvest, and circles showed the percentage of variation explained by each factor alone. The percentages of variation explained by the interactions between two of the factors are shown in the squares on the sides. The unexplained variation is depicted in the squares on the bottom. CKOF: organic fertilizer was amended in non-fumigated soil; CKBF: bio-organic fertilizer was amended in non-fumigated soil; FOF: organic fertilizer was amended in fumigated soil; FBF: bio-organic fertilizer was amended in fumigated soil.

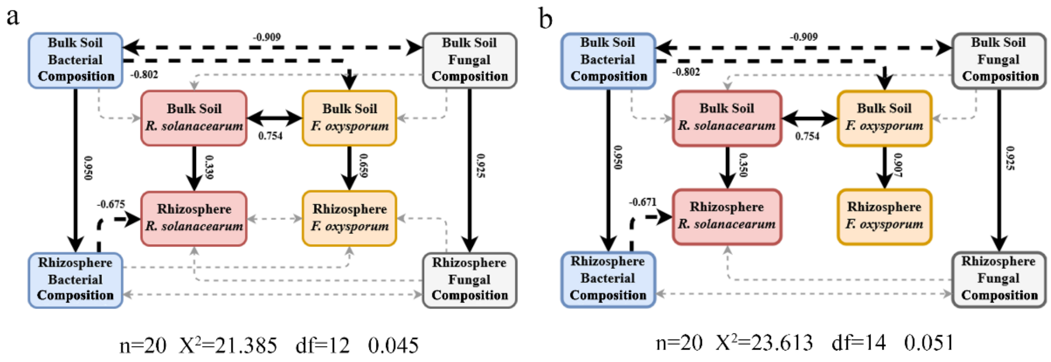

Supplementary Fig 8 Structure equation model of the direct and indirect pathways influencing *R. solanacearum*, *F. oxysporum* and disease incidence. These two incomplete model show the process of production of Fig 6c. Solid and dotted lines represent statistically significant ($P \leq 0.05$) and non-significant relationships, respectively. The path coefficients associated with each arrow of significant relationships are shown.

Supplementary Methods for Supplementary Fig 9: The co-occurrence network was calculated through random matrix theory (RMT)-based approach in MENA online pipeline (http://ieg4.rccc.ou.edu/mena/) [7]. Only the OTUs detected in more than 80% samples and the relative abundance were more than 0.01% were selected to calculate the Spearman rank correlation matrix. And we calculated two networks for bulk and rhizosphere bacteria and pathogens. The cutoff r value was determined to be 0.52 for bulk and 0.53 for rhizosphere. Topological properties of the networks were computed and visualized by Gephi (version 0.9.2).

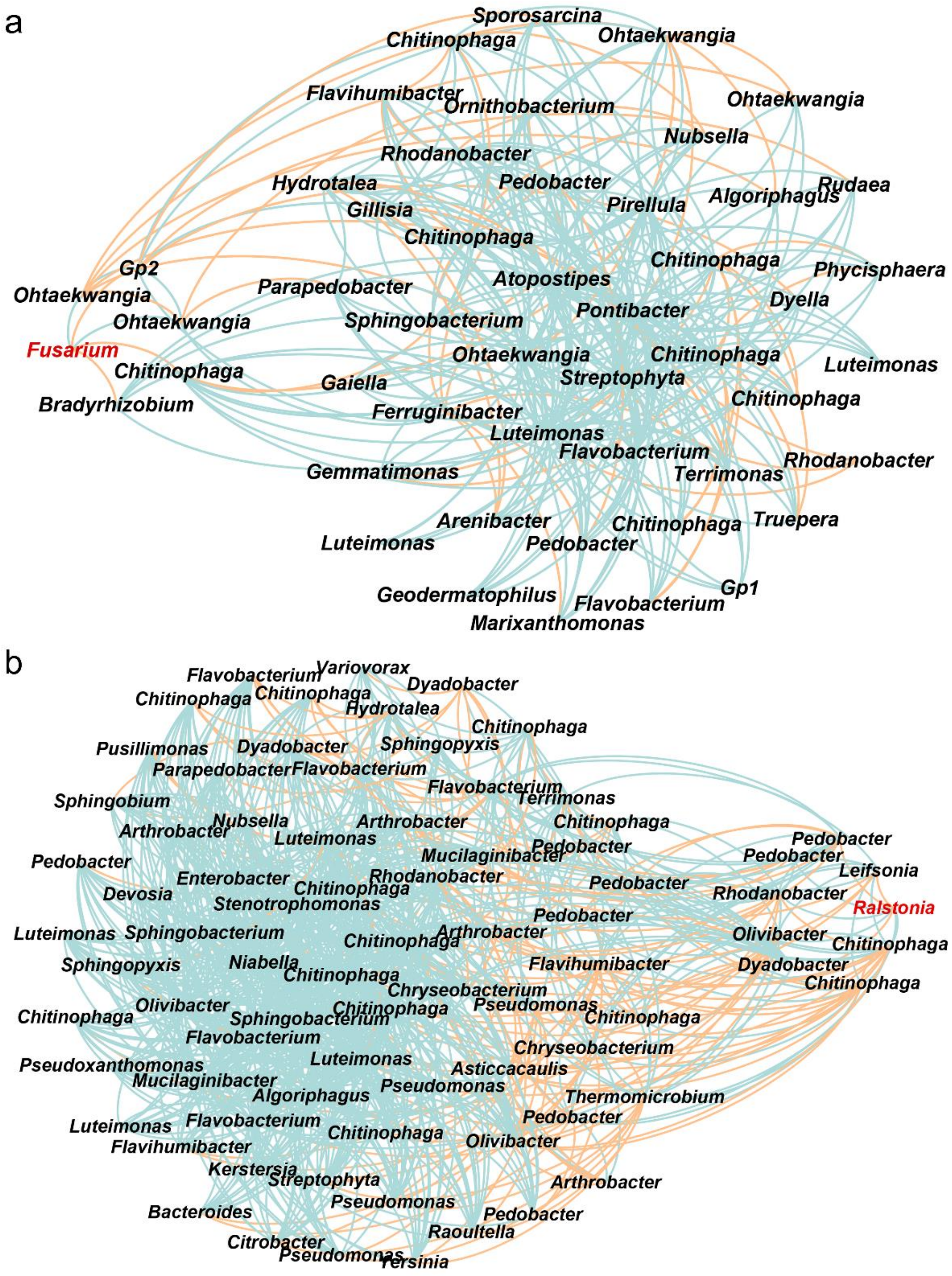

Supplementary Fig 9 Co-occurrence networks for bulk (a) and rhizosphere (b) bacterial OTUs (relative abundance > 0.1%) and pathogens. Yellow lines represent positive correlation, green lines represent negative correlation, and pathogens are shown in red.

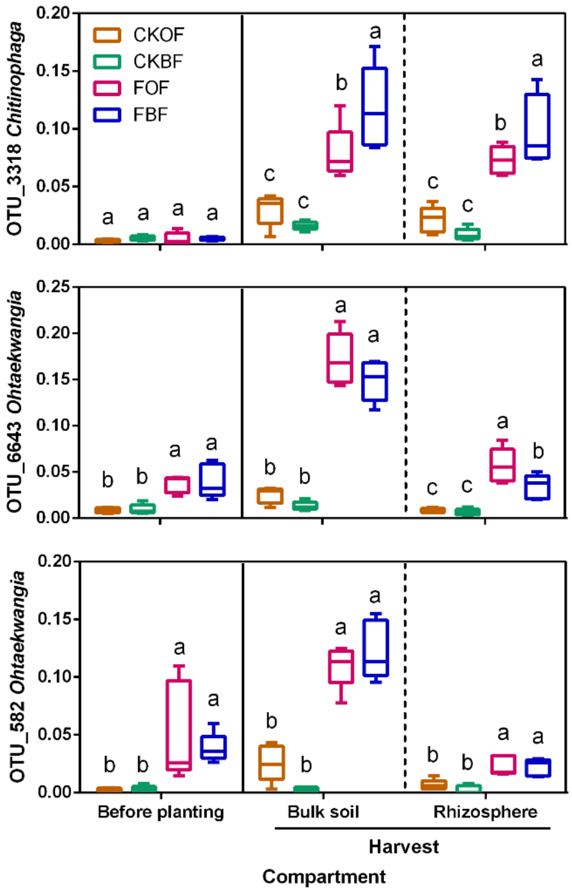

Supplementary Fig 10 Square root of relative abundances of bacterial taxa showing significant correlations with *F. oxysporum* abundance. CKOF: organic fertilizer was amended in non-fumigated soil; CKBF: bio-organic fertilizer was amended in non-fumigated soil; FOF: organic fertilizer was amended in fumigated soil; FBF: bio-organic fertilizer was amended in fumigated soil. The letters indicate significant differences among treatments as determined by Tukey's test ($p<0.05$).

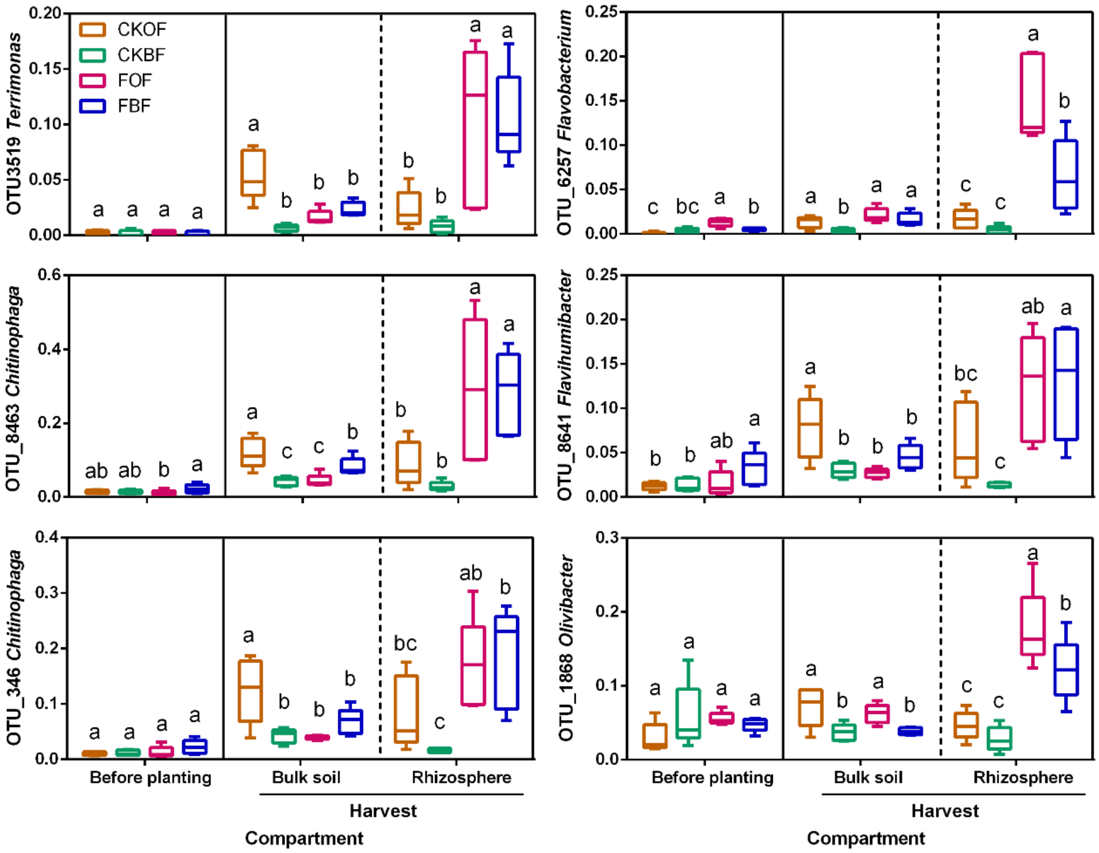

Supplementary Fig 11 Square root of relative abundances of bacterial taxa showing significant correlations with *R. solanacearum* abundance. CKOF: organic fertilizer was amended in non-fumigated soil; CKBF: bio-organic fertilizer was amended in non-fumigated soil; FOF: organic fertilizer was amended in fumigated soil; FBF: bio-organic fertilizer was amended in fumigated soil. The letters indicate significant differences among treatments as determined by Tukey's test ($p<0.05$).

Supplementary Methods for Supplementary Fig 12: For testing the interaction between *Ralstonia solanacearum* and *Fusarium oxysporum* f. sp. *Lycopersici*, we carried out a verification experiment. Two kinds of farmland soils, one was cultured by long-term application of chemical fertilizer (CF) and another was cultured by long-term application of organic fertilizer (OF), were used for this experiment. And two treatments were set: 1. Rs: 10^6 cells/ml *R. solanacearum* were inoculated in soils; 2. Rs+FoL: 10^6 cells/ml *R. solanacearum* and 10^6 spores/ml *F. oxysporum* f. sp. *Lycopersici* were inoculated in soils. Then we planted tomatoes in these soils. Finally, we measured 3 replicates of abundance of *R. solanacearum* in bulk soil and rhizosphere in each kind of soil of each treatment after one month planting by culturable method. The results showed that *F. oxysporum* could promote the abundance of *R. solanacearum* in soils. Thus, the information we got from statistical analysis is verified in this experiment.

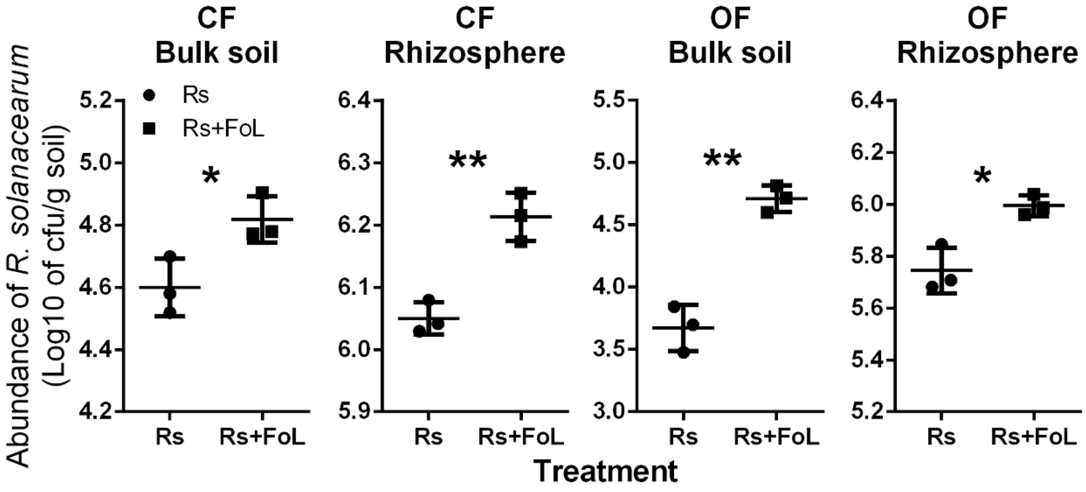

Supplementary Fig 12 Abundance of *Ralstonia solanacearum* in bulk soil and rhizosphere. CF: soils with long-term application of chemical fertilizer, OF: soils with long-term application of organic fertilizer; Rs: 10^6 cells/ml *Ralstonia solanacearum* were inoculated in soils, Rs+FoL: 10^6 cells/ml *Ralstonia solanacearum* and 10^6 spores/ml *Fusarium oxysporum* f. sp. Lycopersici were inoculated in soils. Bars with p value among the two treatments was defined by t-test (*p<0.05, **p<0.01)

Supplementary Scripts:

###The script details of the UPARSE pipeline:

```
usearch -fastq_mergepairs *_R1.fq -fastqout merged.fq    -relabel @
usearch -fastq_filter merged.fq -fastq_trunclen 200 -fastq_maxee 0.5 -fastaout filtered.fa
usearch -fastx_uniques filtered.fa -fastaout uniques.fa -sizeout -relabel Uniq
usearch -cluster_otus uniques.fa -otus otus.fa -uparseout out.up -relabel OTU -minsize 2
usearch -otutab merged.fq -otus otus.fa -otutabout otutab_raw.txt
```

###The script of AMOVA in MOTHUR.

```
sub.sample(shared=otu.shared,size=42254)      # 42254 is the data that your data will be rarefied to.
dist.shared(shared= otu.0.03.subsample.shared,calc=jclass-thetayc-braycurtis)
amova(phylip=otu.0.03.subsample.braycurtis.0.03.lt.dist, design=BAC.design)
```

###The script of PCoA and NMDS in MOTHUR.

```
pcoa(phylip=allfunga.0.03.subsample.braycurtis.0.03.lt.dist)
nmds(phylip=wangfun.0.03.subsample.braycurtis.0.03.lt.dist)
```

###MRT

```
library(mvpart)
library(MVPARTwrap)
spe3 <- read.csv("2015bacmrt.csv", row.names=1)      # input OTUs data
env3 <- read.csv("2015bacenv.csv", row.names=1)      # input environmental data
spe.norm <- decostand(spe3, "normalize")
env3 <- read.csv("mrtenv.csv", row.names=1)
spe.ch.mvpart <- mvpart(data.matrix(spe.norm) ~ ., env3, margin=0.08, cp=0, xv="pick",
xval=nrow(spe3), xvmult=100)
spe.ch.mvpart.wrap <- MRT(spe.ch.mvpart, percent=10, species=colnames(spe3))
summary(spe.ch.mvpart.wrap)
```

###VPA

```
library(vegan)
OTU=read.csv("itst.csv",h=T,row.names=1)      # input OTUs data
env=read.csv("env.csv",h=T,row.names=1)      # input environmental data or treatment data
mm1=model.matrix(~Fumigation,env)[,-1]
mm2=model.matrix(~Fertilizer,env)[,-1]
otu.hel=decostand(OTU,"hel")
mode=varpart(otu.hel,mm1,mm2)
mode
```

###SEM

```
library(sem)
library(dplyr)
```

```
library(DiagrammeR)
library(lavaan)
library(semPlot)
env=read.table("SEM.txt",header = TRUE)
model <- '
DI~RRS+RFOL+RB+RF                          # "~": regressions
RRS~BRS+RB+RF+BB+BF
RFOL~BFOL+RB+RF+BB+BF
BRS~BB+BF
BFOL~BB+BF
RB~BB
RF~BF
BB~~BF                                     # "~~":    correlations
RB~~RF
RRS~~RFOL
BRS~~BFOL
'
fit <- sem(model, data=env)
summary(fit, standardized=TRUE)
```

Supplementary reference:

1. Fierer, N., Jackson, J. A., Vilgalys, R. & Jackson, R. B. Assessment of soil microbial community structure by use of taxon-specific quantitative PCR assays. *Appl. Environ. Microbiol.* **71**, 4117–20 (2005).
2. Schönfeld, J., Heuer, H., Van Elsas, J. D. & Smalla, K. Specific and sensitive detection of Ralstonia solanacearum in soil on the basis of PCR amplification of fliC fragments. *Appl. Environ. Microbiol.* **69**, 7248–7256 (2003).
3. Inami, K. *et al.* Real-time PCR for differential determination of the tomato wilt fungus, Fusarium oxysporum f. sp. lycopersici, and its races. *J. Gen. Plant Pathol.* **76**, 116–121 (2010).
4. Bates, S. T. *et al.* Examining the global distribution of dominant archaeal populations in soil. *ISME J.* **5**, 908–917 (2011).
5. Gardes, M. & Bruns, T. D. ITS primers with enhanced specificity for basidiomycetes **-** application to the identification of mycorrhizae and rusts. *Mol. Ecol.* **2**, 113–118 (1993).
6. White, T. *et al.* Amplification and direct sequencing of fungal ribosomal RNA genes for phylogenetics. *PCR Protoc. A Guid. to methods Appl.* **18**, 315–322 (1990).
7. Deng, Y. *et al.* Molecular ecological network analyses. *BMC Bioinformatics* **13**, 1–20 (2012).
