# Supplementary material for: Soil microbiome manipulation triggers direct and possible indirect suppression against Ralstonia solanacearum and Fusarium oxysporum
Source: NPJ Biofilms Microbiomes. 2021 Apr 12;7:33. doi: 10.1038/s41522-021-00204-9 (PMC8041757; doi:10.1038/s41522-021-00204-9)
Supplement: Supplementary file 2 — Reporting Summary [file 41522_2021_204_MOESM2_ESM.pdf]

nature research

NPJBIOFILMS-00871R3
Corresponding author(s): Rong Li
Last updated by author(s): 2021/03/04

Reporting Summary

Nature Research wishes to improve the reproducibility of the work that we publish. This form provides structure for consistency and transparency in reporting. For further information on Nature Research policies, see our Editorial Policies and the Editorial Policy Checklist.

Statistics

For all statistical analyses, confirm that the following items are present in the figure legend, table legend, main text, or Methods section.

n/a	Confirmed	
☐	☒	The exact sample size (*n*) for each experimental group/condition, given as a discrete number and unit of measurement
☐	☒	A statement on whether measurements were taken from distinct samples or whether the same sample was measured repeatedly
☐	☒	The statistical test(s) used AND whether they are one- or two-sided<br>*Only common tests should be described solely by name; describe more complex techniques in the Methods section.*
☐	☒	A description of all covariates tested
☐	☒	A description of any assumptions or corrections, such as tests of normality and adjustment for multiple comparisons
☐	☒	A full description of the statistical parameters including central tendency (e.g. means) or other basic estimates (e.g. regression coefficient) AND variation (e.g. standard deviation) or associated estimates of uncertainty (e.g. confidence intervals)
☐	☒	For null hypothesis testing, the test statistic (e.g. *F*, *t*, *r*) with confidence intervals, effect sizes, degrees of freedom and *P* value noted<br>*Give P values as exact values whenever suitable.*
☒	☐	For Bayesian analysis, information on the choice of priors and Markov chain Monte Carlo settings
☐	☒	For hierarchical and complex designs, identification of the appropriate level for tests and full reporting of outcomes
☐	☒	Estimates of effect sizes (e.g. Cohen's *d*, Pearson's *r*), indicating how they were calculated

Our web collection on statistics for biologists contains articles on many of the points above.

Software and code

Policy information about availability of computer code

Data collection

Amplicons were sequenced using the Illumina Miseq PE250 platform at the Personal Biotechnology Co., Ltd, Shanghai, China.

Data analysis

```
###The script details of the UPARSE pipeline:
usearch -fastq_mergepairs *_R1.fq -fastqout merged.fq  -relabel @
usearch -fastq_filter merged.fq -fastq_trunclen 200 -fastq_maxee 0.5 -fastaout filtered.fa
usearch -fastx_uniques filtered.fa -fastaout uniques.fa -sizeout -relabel Uniq
usearch -cluster_otus uniques.fa -otus otus.fa -uparseout out.up -relabel OTU -minsize 2
usearch -otutab merged.fq -otus otus.fa -otutabout otutab_raw.txt

###The script of AMOVA in MOTHUR.
sub.sample(shared=otu.shared,size=42254)   # 42254 is the data that your data will be rarefied to.
dist.shared(shared= otu.0.03.subsample.shared,calc=jclass-thetayc-braycurtis)
amova(phylip=otu.0.03.subsample.braycurtis.0.03.lt.dist, design=BAC.design)

###The script of PCoA and NMDS in MOTHUR.
pcoa(phylip=allfunga.0.03.subsample.braycurtis.0.03.lt.dist)
nmds(phylip=wangfun.0.03.subsample.braycurtis.0.03.lt.dist)

###MRT
library(mvpart)
library(MVPARTwrap)
spe3 <- read.csv("2015bacmrt.csv", row.names=1)  # input OTUs data
env3 <- read.csv("2015bacenv.csv", row.names=1)  # input environmental data
```

```
spe.norm <- decostand(spe3, "normalize")
env3 <- read.csv("mrtenv.csv", row.names=1)
spe.ch.mvpart <- mvpart(data.matrix(spe.norm) ~ ., env3, margin=0.08, cp=0, xv="pick", xval=nrow(spe3), xvmult=100)
spe.ch.mvpart.wrap <- MRT(spe.ch.mvpart, percent=10, species=colnames(spe3))
summary(spe.ch.mvpart.wrap)

###VPA
library(vegan)
OTU=read.csv("itst.csv",h=T,row.names=1)   # input OTUs data
env=read.csv("env.csv",h=T,row.names=1)   # input environmental data or treatment data
mm1=model.matrix(~Fumigation,env)[,-1]
mm2=model.matrix(~Fertilizer,env)[,-1]
otu.hel=decostand(OTU,"hel")
mode=varpart(otu.hel,mm1,mm2)
mode

###SEM
library(sem)
library(dplyr)
library(DiagrammeR)
library(lavaan)
library(semPlot)
env=read.table("SEM.txt",header = TRUE)
model <- '
DI~RRS+RFOL+RB+RF          # "~": regressions
RRS~BRS+RB+RF+BB+BF
RFOL~BFOL+RB+RF+BB+BF
BRS~BB+BF
BFOL~BB+BF
RB~BB
RF~BF
BB~~BF                     # "~~":  correlations
RB~~RF
RRS~~RFOL
BRS~~BFOL
'
fit <- sem(model, data=env)
summary(fit, standardized=TRUE)
```

For manuscripts utilizing custom algorithms or software that are central to the research but not yet described in published literature, software must be made available to editors and reviewers. We strongly encourage code deposition in a community repository (e.g. GitHub). See the Nature Research guidelines for submitting code & software for further information.

Data

Policy information about availability of data

All manuscripts must include a data availability statement. This statement should provide the following information, where applicable:

- Accession codes, unique identifiers, or web links for publicly available datasets
- A list of figures that have associated raw data
- A description of any restrictions on data availability

The raw sequence data for the 16S rRNA gene and the ITS region of all samples were submitted to the NCBI Sequence Read Archive database (https://www.ncbi.nlm.nih.gov/) with the accession number SRP188824.

Field-specific reporting

Please select the one below that is the best fit for your research. If you are not sure, read the appropriate sections before making your selection.

☐ Life sciences ☐ Behavioural & social sciences ☒ Ecological, evolutionary & environmental sciences

For a reference copy of the document with all sections, see nature.com/documents/nr-reporting-summary-flat.pdf

Ecological, evolutionary & environmental sciences study design

All studies must disclose on these points even when the disclosure is negative.

Study description	Regulating soil ecosystem services through microbiome manipulation represents an innovative strategy to maintain soil functioning. For instance, a soil microbiome manipulation approach could potentially reduce the use of pesticides by improving the ability of soils to resist or recover from pathogen infestation, thus generating natural suppressiveness. Here we investigated the extent to which disturbance could modify the soil microbiome in order to suppress the pathogens Ralstonia solanacearum and Fusarium oxysporum in tomato monoculture. For that we simulated disturbance through soil fumigation and investigated how the subsequent application with bio-organic and organic amendment re-shapes the taxonomic and functional potential of the soil microbiome. The use of organic amendment alone generated smaller shifts in bacterial and fungal community composition and no suppressiveness,

fumigation directly decrease F. oxysporum and induced drastic changes in the soil microbiome. This was further converted from a disease conducive to a suppressive soil microbiome due to application of organic amendment, which affected the way the bacterial and fungal communities were reassembled. These direct and possible indirect effects resulted in a high-efficient disease control rate, providing a promising strategy for control of the disease caused by multiple pathogens. Collectively, this study provides an integrative approach and a set of agricultural practices that disentangled the microbiological and ecological principles driving soil microbiome suppressiveness to pathogens.

Research sample	Field experiment was performed in Hengxi town in Nanjing, Jiangsu province (32°02′N, 118°50′E) which has a tropical monsoon climate with an average annual temperature and precipitation of 15.4 °C and 1106 mm, respectively. The experiment was performed for three field seasons, continuously, from March 2014 to June 2015, with two seasons in 2014 and one in 2015. The field experiment consisted of 4 treatments: CKOF, 0.3 kg/m2 organic fertilizer (OF) was amended in un-fumigated (CK) soil; CKBF, 0.3 kg/m2 bio-organic fertilizer (BF) was amended in un-fumigated soil; FOF, 0.3 kg/m2 organic fertilizer was amended in fumigated (F) soil; and FBF, 0.3 kg/m2 bio-organic fertilizer was amended in fumigated soil. Each treatment had three randomized independent replications and each replicate contained 40 tomato plants. The tomato variety used in this experiments was "Shi Ji Fen Guan", which is one of the early ripening tomato cultivars with pink round big fruit, is normally cropped in this area and susceptible to soil-borne disease 17. Fumigation was achieved by mixing 0.15 kg/m2 ammonium bicarbonate and 0.15 kg/m2 lime, and after application of fumigant, all treatments were covered with plastic film for 15 days before fertilization. Fertilization consisted of applying chicken manure compost (N: 2.0%, P: 0.9%, K: 0.9%) or bio-organic fertilizer (N: 2.2%, P: 1.0%, K: 1.0%) to treatments OF and BF, respectively. We compensated the nutrient differences between two types of fertilizers with mineral fertilizer. Bio-organic fertilizer was produced by inoculation of Bacillus amyloliquefaciens T-5 26 into an organic mixture of rapeseed meal and chicken manure composts at a ratio of 1:4 (dw/dw) for the solid fermentation process.
Sampling strategy	Soil samples were collected before planting and during harvest in 2015. Samples collected at the 7th day after fumigation when before fertilization in March were defined as before planting, and bulk and rhizosphere soil samples collected during harvest in June were defined as harvesting period samples. For bulk soil sample collection before planting, in brief, a nine-point sampling method was utilized to collect soil cores with 0-15 cm depth from the surface in each plot to form a composite sample, and we did twice nine-point sampling in each plot to form two composite samples as two replicates so that we had six bulk soil replicates in each treatment. And for bulk soil sample collection at harvest, we collect nine soil cores with 0-15 cm depth from the surface after removing plants in each plot to form a composite sample as one replicate, and we sampled two replicates in each plot as well as before planting. All bulk soil samples in each replicate was subsequently mixed individually in a 2-mm sieve to homogenize the soil, one portion of each sample was stored at -80oC for further DNA extraction, and the other portion was air dried for chemical analyses. All bulk soil chemical properties were determined according to Liu et al (2018). For rhizosphere sample, two tomato roots were collected from each plot, shaken vigorously to remove excess soil, and then the soil adhering to the roots (rhizosphere soil) was removed by sterile water. We collected two rhizosphere samples from each plot, which were frozen stored at −80°C for soil DNA extraction. Thus, 6 bulk and 6 rhizosphere soil samples were collected for each treatment.
Data collection	In each season, disease incidence was recorded when most of the tomato fruits were ripe. The symptoms caused by R. solanacearum are wilted leaves maintain green color and the vascular tissues in the lower stem of wilted plants show a dark brown discoloration. The symptoms caused by F. oxysporum f. sp. Lycopersici were older leaves droop, curve downward, turning yellow and the vascular tissue of a diseased plant is dark brown 27. In this study, diseased tomato showed bacterial and Fusarium wilt symptoms simultaneously. So we defined diseased plant based on observations of typical wilt symptoms 8,28, for instance, necrosis and drooping of the leaves. Disease incidence of the field experiment was calculated by counting the number of tomato plants with wilt symptom among the total number of plants, each treatment of each season have three replicates.<br>Five bulk and rhizospheric soil samples from each treatment and period were randomly chosen for subsequent DNA extraction using the PowerSoil Soil DNA Isolation Kits (MoBio Laboratories Inc., USA) following the manufacturer's protocol. The concentration and quality of DNA were measured through a spectrophotometer (NanoDrop 2000, USA) to ensure that the DNA is available for subsequent analysis. The abundance of bacteria, fungi, Ralstonia solanacearum and Fusarium oxysporum f. sp. Lycopersici were quantified by quantitative PCR (qPCR) with primers described in table S1. The qPCR analyses were carried out with an Applied Biosystems 7500 real-time PCR system (Applied Biosystems, CA) using SYBR green I fluorescent dye detection in 20-µl volumes containing 10 µl of SYBR Premix Ex Taq (TaKaRa Bio Inc., Japan), 2 µl of template, and 0.4 µl of both forward and reverse primers (10 mM each). All qPCR reactions were performed using the standard temperature profile (Hu et al., 2016). Each sample was performed in three replicates, and the results were expressed as log10 values (target copy number g−1 soil).<br>The bacterial 16S rRNA gene V4 region was amplified from soil genomic DNA by primers 520F and 802R, while ITS1f and ITS2 were used for amplification of the fungal internal transcribed spacer 1 (ITS1) region (primers are described in table S1). Amplicons were sequenced using the Illumina Miseq PE250 platform at the Personal Biotechnology Co., Ltd, Shanghai, China.
Timing and spatial scale	The experiment was performed for three field seasons, continuously, from March 2014 to June 2015, with two seasons in 2014 and one in 2015.
Data exclusions	no data exclusions in this study.
Reproducibility	We repeat this field trial three times, so the consequence can be reproduced.
Randomization	This experiment had four treatments, each treatment had three randomized independent replications and each replicate contained 40 tomato plants.
Blinding	To minimize the effect of personal observation, the diseased plants were counted twice by two persons, separately.

Did the study involve field work? ☒ Yes ☐ No

Field work, collection and transport

Field conditions	This place has a tropical monsoon climate with an average annual temperature and precipitation of 15.4 °C and 1106 mm, respectively.

Location	Field experiment was performed in Hengxi town in Nanjing, Jiangsu province (32°02′N, 118°50′E)
Access & import/export	This field, which belong to Nanjing institution of vegetable and flower science, is not far (40 km) with our lab. we rent the field for this trial and the soil samples were not hazardous. So it is not necessary to get access for import or export.
Disturbance	this field trail was performed in a plastic green house, so that it could minimize the disturbance of environment.

Reporting for specific materials, systems and methods

We require information from authors about some types of materials, experimental systems and methods used in many studies. Here, indicate whether each material, system or method listed is relevant to your study. If you are not sure if a list item applies to your research, read the appropriate section before selecting a response.

Materials & experimental systems

n/a	Involved in the study
☒	☐ Antibodies
☒	☐ Eukaryotic cell lines
☒	☐ Palaeontology and archaeology
☒	☐ Animals and other organisms
☒	☐ Human research participants
☒	☐ Clinical data
☒	☐ Dual use research of concern

Methods

n/a	Involved in the study
☒	☐ ChIP-seq
☒	☐ Flow cytometry
☒	☐ MRI-based neuroimaging
